# Breastfeeding counselling mentorship programme feasibility: a mixed-methods study

**DOI:** 10.1017/S1368980025100591

**Published:** 2025-07-04

**Authors:** Brian Micino Njoroge, Sascha Lamstein, Kathryn Beck, Jackline A. Odhiambo, Silvia Alayon, Beatrice C. Mutai, Esther Mogusu, Josephine Wandia Munene, James Kanyuira Njiru, Susan A. Were, Delaney Ward, Iscah Achieng Okello, Julie Koroso, Caroline K. Arimi, Florence Mugo

**Affiliations:** 1 USAID Advancing Nutrition, Nairobi, Kenya; 2 JSI Research & Training Institute, Inc., Boston, USA; 3 Mbagathi County Referral Hospital, Nairobi City County, Nairobi, Kenya; 4 University of Nairobi, Faculty of Health Sciences, Department of Paediatrics and Child Health, Nairobi, Kenya; 5 Save the Children, Nairobi, Kenya; 6 Kenya Association for Breastfeeding, Nairobi, Kenya; 7 Ministry of Health Division of Family Wellness, Nutrition and Dietetics, Nairobi, Kenya; 8 Department of Health, Wellness and Nutrition, Nairobi City County, Nairobi, Kenya; 9 Nyanam Widows Rising, Kisumu, Kenya; 10 Independent Consultant; 11 Save the Children, Fairfield, USA

**Keywords:** Breastfeeding counselling, Mentorship, Feasibility, Capacity strengthening

## Abstract

**Objectives:**

To determine the feasibility of implementing a facility-based breastfeeding counselling (BFC) mentorship programme and its effect on mentee confidence and client perceptions of BFC.

**Setting:**

Mbagathi County Referral Hospital in Nairobi, Kenya.

**Participants:**

Health facility management, health workers (twenty-one mentees and seven mentors), 120 pregnant women in the third trimester who attended an antenatal care (ANC) appointment at Mbagathi Hospital and reported receiving BFC during a visit in the 2 weeks prior and 120 postpartum women in the postnatal care ward who delivered a full-term infant and reported receiving BFC.

**Design:**

Mixed-methods study incorporating online surveys, client exit interviews, key informant interviews and focus group discussions. The 4-month intervention involved facility-wide orientations, selection and training of mentors, assigning mentees to mentors and implementing mentorship activities.

**Results:**

The programme successfully maintained 90·5 % mentee retention (19/21) over 4 months. At baseline, mentees demonstrated high knowledge (94 % questions answered correctly), which was maintained at endline (92 %). Mentees showed significant improvement in confidence counselling on breastfeeding and infant feeding (67 % at baseline *v*. 95 % at endline, *P* = 0·014). The percentage of ANC clients who felt BFC gave them more knowledge increased from 73 % to 97 % (*P* < 0·001). Among postnatal care clients, those reporting friendly treatment increased from 89 % to 100 % (*P* = 0·007), verbal mistreatment declined from 7 % to 0 % (*P* = 0·044) and those feeling discriminated decreased from 11 % to 2 % (*P* = 0·03). Key enablers included administrative support, structured mentorship tools and peer learning communities. Implementation barriers included scheduling conflicts, staff shortages and high patient volumes.

**Conclusions:**

BFC mentorship was feasible in this setting and was associated with improved health worker confidence in BFC. The programme can be successfully implemented with supportive facility leadership, well-matched mentors and mentees and adaptable mentorship approaches.

Breastfeeding is critical for child survival. Promotion of early initiation and exclusive breastfeeding for 6 months with continued breastfeeding for up to 24 months is one of ten interventions that, if implemented at 90 % coverage, could reduce child mortality by 15 %^(1)^. Breastfeeding prevents major causes of newborn and child mortality and may reduce the risk of childhood obesity and type 2 diabetes later in life^(2–7)^. For mothers, breastfeeding protects against breast and ovarian carcinoma and reduces the risk of type 2 diabetes^(8)^. Furthermore, breastfeeding is associated with improved performance in intelligence tests among children^(9)^, while breastfeeding is not associated with ‘economic losses of about $302 billion annually’^(10)^.

Early initiation has been shown to improve exclusive breastfeeding rates^(4)^. However, globally, less than half of all newborns are put to the breast within 1 h of birth, and only two out of five infants under 6 months of age are exclusively breastfed^(4)^. In Kenya, 60 % of children are put to the breast within 1 h of birth, and 60 % of infants aged 0–5 months are exclusively breastfed^(11)^. The prevalence of exclusive breastfeeding in Kenya has remained largely unchanged since 2014^(11)^.

Breastfeeding education^(12)^, counselling and support can improve breastfeeding practices^(13–15)^. It has been demonstrated that breastfeeding counselling (BFC) can result in a 90 % increase in exclusive breastfeeding of infants aged 0–5 months. A 2015 meta-analysis found that counselling or education increased the rates of early initiation, exclusive breastfeeding and continued breastfeeding, particularly in low- and middle-income countries^(16)^. In Kenya, 87 % of children whose mothers received BFC during antenatal care (ANC) visits were exclusively breastfed for the first 2 d compared with 27 % of children whose mothers did not receive BFC during ANC^(11)^. While scaling BFC as part of routine ANC and postnatal care (PNC) could improve breastfeeding practices^(17)^, BFC is not well integrated into the health system in Kenya^(18)^.

Effective BFC requires skilled ANC and PNC providers. The 2018 WHO *Guideline: Counselling of Women to Improve Breastfeeding Practices* recommends that all pregnant women and mothers with young children receive BFC at least six times from the antenatal period through age 2^(19)^. Step 2 of the Baby-Friendly Hospital Initiative (BFHI) Ten Steps focuses on ensuring staff have sufficient skills to support breastfeeding^(7)^. One way to build health worker competencies is through the *BFHI Training Course for Maternity Staff* (BFHI training)^(20)^.

The Kenya Ministry of Health (MoH) prioritised capacity strengthening for quality maternal, infant and young child nutrition service delivery^(21)^. Mentoring interventions, in addition to training, have been found effective in strengthening the capacity of health workers and in improving the clinical management of infectious diseases among mothers, newborns and children^(22–24)^. Mentoring interventions may also increase health workers’ adherence to guidelines, standards and protocols^(23)^. Additionally, mentorship programmes have been shown to strengthen health workers’ confidence and ability to implement a range of practices^(25)^.

In 2022, stakeholders in Kenya proposed the development of a facility-based mentorship programme to strengthen health workers’ BFC competencies. The Division of Nutrition and Dietetics of the Kenya MoH; the BFHI Task Force of the Maternal, Infant and Young Child Nutrition technical working group and USAID Advancing Nutrition developed *Implementation Guidance for a Facility-Based Breastfeeding Counselling Mentorship Program* ^(26)^, leveraging global guidance and tools, including the BFHI training^(20)^ and the *Competency Verification Toolkit: Ensuring Competency of Direct Care Providers to Implement the BFHI* ^(27)^ to design the facility-based implementation guidance. The Implementation Guidance provides a structured framework for establishing a facility-based BFC mentorship programme. It outlines specific roles and responsibilities for different stakeholders – from facility leadership to individual mentors and mentees – and provides adaptable tools such as mentee self-assessment tool, mentor observation checklists and mentor feedback forms. The *Competency Verification Toolkit* observation tools informed our verification approach for assessing knowledge and building health workers’ BFC skills across sixteen specific competencies necessary for implementing the Ten Steps to Successful Breastfeeding. Our study prioritised competencies in foundational skills; antenatal period, birth and immediate postpartum; essential issues for breastfeeding mothers and care at discharge. These resources were designed to be adaptable while maintaining fidelity to global BFHI standards, allowing for contextual implementation across different facility types and resource levels.

In 2023, the Kenya MoH and USAID Advancing Nutrition conducted this study to assess the feasibility of the BFC mentorship programme in the ANC and PNC departments of Mbagathi County Referral Hospital (Mbagathi Hospital). This study aimed to identify factors that enabled and hindered the programme’s implementation and measure its impact on health workers’ confidence and clients’ perceptions of BFC.

## Methods

### Study description

#### Study setting

The study was conducted in the ANC and PNC departments of Mbagathi Hospital, a level five public county referral hospital located in Kibra Sub-County, Nairobi, Kenya.

We selected this health facility in consultation with stakeholders for several reasons. First, the hospital has a high patient load; per day, it serves 75–80 pregnant women through the ANC clinic and has 25–30 live births^(28)^. This large number of deliveries serves as an indicator of the high need for skilled BFC in the facility. Second, at the time, health facility staff had not been trained in the BFHI. This provided an opportunity for the study team to deliver the BFHI training pre-intervention as a prerequisite to the BFC mentorship programme. Finally, Mbagathi Hospital has maternal and child health programmes that complement the BFC mentorship programme, including a kangaroo mother care unit, staff trained on emergency obstetric and newborn care and continuous quality improvement teams.

The ANC and PNC departments were chosen as the focus of the study because of the importance of providing timely BFC during prenatal care and immediately after delivery. Additionally, it was important to ensure that our results would be useful in informing BFC implementation interventions within other public-level five referral hospitals in Kenya.

#### Description of the intervention

The intervention was a facility-based mentorship programme carried out from March to September 2023 in accordance with the implementation guidance for a facility-based BFC mentorship programme^(26)^. This implementation guidance serves as a bridge between the BFHI Training Course and the BFHI Competency Verification Toolkit. It provides a comprehensive background on BFC, programme rationale and a programme management structure specifically designed for facility-based BFC mentorship, along with monitoring systems and adaptable tools for mentors and mentees.

For the study, we prioritised BFC competencies in foundational skills: communicating in a credible, effective way; antenatal, birth and immediate postpartum care; essential issues for breastfeeding mothers and care at discharge.

Prior to programme implementation, a comprehensive stakeholder sensitisation process was conducted. This included meetings with the Chief Executive Officer, Hospital Management Team, Reproductive Health department heads and Hospital administrator to secure institutional buy-in. The sensitisation emphasised the programme’s alignment with national breastfeeding promotion policies and its potential benefits for maternal and child health care, which was crucial for gaining administrative support and facilitating integration into existing hospital workflows.

After a 4-day training for ANC and PNC department health workers on the BFHI, implementation began with establishing the mentorship programme leadership structure at the facility. National BFHI training facilitators, the BFHI coordinator and the mentorship coordinator selected mentors (see Study Participants section below). Mentors, the facility BFHI coordinator and the facility mentorship coordinator participated in a 2-day *Core Concepts in Mentorship Training for the Breastfeeding Counselling Mentorship Program* ^(29)^ – an evidence-based curriculum grounded in adult learning principles that covers interpersonal communication, clinical teaching methodologies and contextual mentoring specific to BFC through participatory exercises aligned with BFHI standards to prepare them to support and guide mentees effectively throughout the programme.

Mentors supported mentees for 4 months. The mentoring involved demonstrations, mentor observations using competency assessment tools and weekly meetings. Meetings were both formal and informal, individual and in small groups. Mentors used clinical teaching, side-by-side mentoring and case presentations. Once per month, mentors, mentees, the BFHI coordinator, the mentorship coordinator and BFHI training facilitators met to review monthly activities, discuss areas for improvement and share experiences.

#### Study design

The study used a mixed-methods approach. The study aimed to identify factors that enabled and hindered the implementation of the facility-based BFC mentorship programme through health worker surveys at baseline and endline and focus group discussions (FGD) and key informant interviews (KII) at endline. We used health worker surveys (at baseline and endline) to measure the effect of the mentorship programme on mentees’ confidence related to BFC. We used client exit interviews (at endline) to determine the perceptions of pregnant and postpartum women related to their BFC experience. While the programme included competency assessments using standardised verification tools, this feasibility study focused primarily on self-reported confidence as a proximal indicator of programme impact. Data from mentors’ observations of counselling skills were collected but will be reported separately. This decision allowed us to prioritise implementation feasibility while maintaining a manageable assessment approach in the busy clinical setting.

#### Study participants and sampling methods

The study involved health workers, pregnant women and postpartum mothers in the ANC and PNC departments of Mbagathi Hospital, as well as health facility leadership. All doctors, nurses, nutritionists, clinical officers and midwives (eighty-seven total) from the ANC and PNC departments at Mbagathi Hospital were considered for participation in the study. Of these, eighty health workers participated in the BFHI training, and from this group, seven mentors and twenty-one mentees were selected for the study. The selection of mentors and mentees (see online supplementary material, Supplemental Material S4) was based on the criteria described in the implementation guidance^(26)^, which includes designation as a doctor, nurse, nutritionist, clinical officer or midwife and at least 2 years of experience in maternal and newborn care. All pregnant women exiting the ANC department and all postpartum women exiting the PNC department on data collection days were screened for eligibility for the study. Eligible ANC clients were in the third trimester (≥ 29 weeks of gestation), had attended an ANC appointment and reported receiving BFC from a health worker during a visit in the prior 2 weeks. For PNC clients, eligibility criteria included having delivered a full-term infant and reported receiving BFC from staff. Enumerators interviewed sixty-two ANC clients at baseline and sixty at endline, and sixty-one PNC clients at baseline and sixty at endline (see Sample Size Calculation below). Health facility leaders purposively selected for KII included members of the health facility management team, the BFHI facility implementation team and the BFHI facility coordinator for Mbagathi Hospital.

#### Sample size calculation for client exit interviews

The required sample size for client exit interviews was calculated based on an estimated 18 live births per day, resulting in approximately 252 live births over each 2-week data collection period – baseline and endline. To achieve a 90 % confidence level with a 10 % margin of error, a sample size of fifty-two per time point per unit/clinic was required. Accounting for a 10 % nonresponse rate, the target sample size was adjusted to 60 per time point per unit/clinic.

The sample size (*n*) and margin of error (*E*) are given by

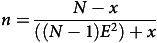




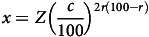




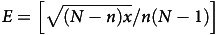

where *N* is the population size, *r* is the fraction of responses of interest and *Z*(*c*/100) is the critical value for the confidence level *c*. The Raosoft sample size calculator was utilised for these calculations.

### Data collection

#### Quantitative data

Health workers completed two online multiple-choice surveys (health worker post-training survey S5) in April 2023 at the start of the mentorship programme (baseline) and in September 2023 after the mentorship programme (endline).

Survey tools were developed based on the Competency Verification Toolkit^(27)^.

Structured client exit interviews were administered by trained enumerators, either in person or by phone, immediately after determining eligibility and obtaining consent (Client Exit Interview Guide S6 and S7). We developed the interview guides in English, using questions in the most recent Service Provision Assessment tool, and had them translated into Kiswahili by a professional translator^(11)^. Enumerators, fluent in both English and Kiswahili, asked clients how often certain actions occurred and how satisfied they were with the BFC services received.

#### Qualitative data

Qualitative data were collected at endline through FGD with mentors and mentees and KII with facility leadership. We conducted two FGD with mentees (one with PNC mentees and one with ANC mentees) and another with mentors. FGD included all mentors and mentees available (six out of seven and fourteen out of twenty-one, respectively). Trained research assistants facilitated FGD and KII using semi-structured guides that explored perceptions of the mentorship programme. FGD and KII were conducted in English. With participants’ consent, they were recorded and subsequently transcribed.

#### Study measures

Health worker surveys included twenty-one knowledge questions, which we computed into an overall test score (number) and percentage of questions answered correctly. Thirty-two questions assessed confidence in their ability to provide quality BFC using a Likert scale (not at all = 0, slightly = 1, somewhat = 2, quite = 3 and extremely = 4), which we converted to a binary outcome of extremely confident or not. We computed the percentage of BFC skills in which the health workers felt extremely confident all the time and also broken down as < 50 %, 50–79 %, and 80–100 % of the time.

Similarly, client exit interviews included twenty-six questions about health worker BFC practices during the ANC visit or postnatally using a Likert scale (no, never = 0; yes, some of the time = 1; yes, most of the time = 2 and yes, all of the time = 3).

### Data analysis

#### Quantitative data

We used Stata v17 for management and analysis of quantitative data (StataCorp LP). We calculated descriptive statistics, numbers and percents for categorical variables and means, medians and interquartile range (IQR) for continuous variables. We conducted bivariate analysis to compare measures across time points, reporting *P*-values from Pearson’s chi-squared test for categorical variables and Wilcoxon rank sum test for continuous variables. For binary outcomes in the paired sample of health worker surveys, we used McNemar’s chi-square test to determine the statistical significance of differences in the proportions observed. We defined statistical significance of differences between time points as *P* < 0·05.

#### Qualitative data

We analysed and coded qualitative data collected during KII and FGD. We developed a coding framework iteratively through deductive and inductive approaches. Using the framework, two people independently coded all the transcripts. They compared results and discussed differences and generated a results matrix in Excel. Due to the limited number of transcripts, we used a rapid matrix-based analysis. The inductive process included familiarisation with data and development of broad codes and their definitions, as well as fine codes with illustrative quotes. All authors reviewed the coding framework and results matrix and helped identify themes. We refined themes through discussion and then interpreted and reported results.

## Results

### Quantitative findings from health worker surveys

#### Characteristics of health workers at baseline

All twenty-one mentees and seven mentors completed the health worker survey at baseline, and nineteen mentees and seven mentors completed it at endline (Table 1). Most health workers were female (76 % of mentees and 86 % of mentors), and 71 % worked in the PNC department. Most mentors and mentees were nurses (76 % and 43 %, respectively). Approximately half of mentees (53 %) had 5 or more years of professional experience, 24 % had 2–4 years and 24 % had less than 2 years. More than half of mentors (57 %) had more than 10 years of experience. At baseline, all mentors and mentees reported having previously counselled clients on breastfeeding. Two mentees did not complete the endline survey. One had transferred to another facility midway through the programme, and the other was out of the country during the endline data collection period. Their baseline data were excluded from the comparative analysis.

**Table 1.** Baseline characteristics of health workers

Health worker characteristics	Mentees		Mentors	
	*n* 21		*n* 7	
	*n*	%	*n*	%
Gender				
Female	16	76 %	6	86 %
Male	5	24 %	1	14 %
Workplace unit				
ANC department	6	29 %	2	29 %
PNC department	15	71 %	5	71 %
Type of health worker				
Nurse/nurse midwife	16	76 %	4	57 %
Nutritionist	5	24 %	3	43 %
Duration of professional work experience (years)				
< 2 years	5	24 %	0	0 %
2–5 years	5	24 %	1	14 %
5–10 years	9	43 %	2	29 %
> 10 years	2	10 %	4	57 %

ANC, antenatal care; PNC, postnatal care.

#### Knowledge and confidence before and after mentorship

Knowledge and confidence related to BFC were high at the start of the programme. Knowledge was retained and confidence remained high throughout the programme (Table 2). On average, mentees answered 94 % of questions correctly at baseline and 92 % at endline. They were extremely confident in their ability to perform 72 % of actions related to BFC competencies at baseline and 75 % at endline. The percentage of mentees who felt extremely confident in their ability to conduct at least 50 % of the BFC actions increased from 71 % to 95 %. In addition, the percentage of mentees who were extremely confident in their ability to counsel women on breastfeeding and infant feeding increased from 67 % to 95 %. We did not observe statistically significant changes in confidence to perform other actions related to BFC. We combined data from multiple sources aligned with the WHO/UNICEF Competency Verification Toolkit. Table 3 presents this synthesis, showing how mentees’ knowledge (based on Annex E multiple-choice knowledge questions), self-reported confidence (aligned with thirty-two performance indicators from observation tools to verify competencies – Annex G) and client-reported experiences interconnect across key competency areas. While knowledge and overall confidence levels were high at baseline and remained stable, specific improvements were observed in counselling confidence, particularly in mentees’ ability to counsel women on breastfeeding and infant feeding, suggesting the mentorship programme reinforced practical application of existing knowledge.

**Table 2.** Mentee knowledge and confidence for counselling pregnant and postpartum women on breastfeeding

Performance indicators	Baseline		Endline		Difference	*P*-value
	*n* 21		*n* 19			
	*n*	%	*n*	%		
Average percent of the knowledge questions that mentees answered correctly	94 %		92 %		−2 %	0·401
Percent of mentees that answered at least 80 % (26) of the knowledge questions correctly	21	100 %	19	100 %	0 %	0·155
Average percent of the BFC actions that mentees felt extremely confident to perform	72 %		75 %		3 %	0·609
Percent of mentees who felt extremely confident in their ability to perform at least 50 % (16) of the BFC actions	15	71 %	18	95 %	24 %	0·155
Health worker felt extremely confident in ability to:						
Use listening and learning skills	14	67 %	10	53 %	–14 %	0·414
Use building confidence and giving support skills	16	76 %	14	74 %	–2 %	0·99
Describe how healthcare practices affect the initiation of breastfeeding	16	76 %	13	68 %	–8 %	0·706
Assess a pregnant woman’s knowledge about breastfeeding	15	71 %	16	84 %	13 %	0·083
Explain the importance of skin-to-skin contact immediately after delivery	16	76 %	17	89 %	13 %	0·180
Explain the importance of initiation of breastfeeding within 1 h after delivery	16	76 %	17	89 %	13 %	0·180
Explain the importance of exclusive breastfeeding for 6 months	16	76 %	17	89 %	13 %	0·180
Explain the benefits of breastfeeding to the mother	14	67 %	17	89 %	22 %	0·059
Explain the benefits of breastfeeding to the child	14	67 %	15	79 %	12 %	0·257
Explain how breastfeeding works	15	71 %	14	74 %	3 %	0·706
Counsel on breastfeeding and infant feeding	14	67 %	18	95 %	28 %	0·014
Explain infant feeding patterns in the first 36 h of life	16	76 %	13	68 %	–8 %	0·706
List the signs and symptoms that indicate a newborn may not be getting enough milk	15	71 %	15	79 %	8 %	0·480
Explain to a mother the signs of adequate transfer of milk in the first few days	15	71 %	14	74 %	3 %	0·706
Explain to a mother the warning signs of infant undernourishment or dehydration	15	71 %	13	68 %	–3 %	> 0·99
Recognise breast refusal and help a mother to breastfeed	13	62 %	13	68 %	6 %	0·739
List the different reasons why a newborn may cry often	16	76 %	14	74 %	–2 %	> 0·99
Explain the importance of continued breastfeeding for up to 2 years and beyond	16	76 %	17	89 %	13 %	0·180
Explain responsive feeding and its implications for the frequency and duration of breastfeeding	15	71 %	14	74 %	3 %	0·564
Assess a breastfeeding session using the job aid: breastfeeding session observation	16	76 %	12	63 %	–13 %	0·414
Help a mother to position her baby for breastfeeding using the four key points of positioning	17	81 %	15	79 %	–2 %	> 0·99
Explain the four key points of attachment for breastfeeding	16	76 %	13	68 %	–8 %	0·706
Help a mother to attach her baby to the breast once they are well-positioned	16	76 %	17	89 %	13 %	0·180
Help a mother who has flat or inverted nipples	15	71 %	15	79 %	8 %	0·480
Help a mother with engorged breasts	14	67 %	12	63 %	–4 %	> 0·99
Help a mother with sore or cracked nipples	15	71 %	14	74 %	3 %	0·706
Help a mother with mastitis	15	71 %	9	47 %	–24 %	0·206
Describe alternative methods of feeding	15	71 %	13	68 %	–3 %	> 0·99
Explain the steps of expressing breast milk by hand	16	76 %	13	68 %	–8 %	0·739
Practice with a mother how to cup feed her baby safely	15	71 %	15	79 %	8 %	0·480
Counsel a mother about her own health	16	76 %	16	84 %	8 %	0·414
Implement the International Code of Marketing of Breast-Milk Substitutes in a health facility	13	62 %	9	47 %	–15 %	0·366

BFC, breastfeeding counselling.

**Table 3.** Summary of change in performance indicators by data source

Performance indicator	Knowledge (mentee survey)	Confidence (mentee survey)	Reported practice (client exit interviews)
11. Demonstrate at least three aspects of listening and learning skills when talking with a mother	Strong: 100 % able to identify an open-ended question at baseline and endline	Reinforcement needed: 67 % were extremely confident in their ability to use listening and learning skills at baseline, only 53 % at endline	Strong: Most clients reported being asked how they were feeling, feeling they could ask questions and being treated in a friendly manner. In addition, most PNC clients reported feeling that the health worker paid attention when they needed help with breastfeeding
15. Engage in a conversation with a pregnant woman on three aspects of the importance of breastfeeding	Strong: 100 % know the risk of not breastfeeding a baby and the importance of breastfeeding for the mother at baseline and endline	Strong: Increase in percentage of participants who were extremely confident in their ability to explain the importance of continued breastfeeding for up to 2 years (76 % and 89 %, respectively), the benefits of breastfeeding to the mother (67 % and 89 %, respectively) and the benefits of breastfeeding to the child (67 % and 79 %, respectively)	Not asked, further investigation needed
16. Assess at least three aspects of a pregnant woman’s knowledge about breastfeeding in order to fill gaps and correct inaccuracies	N/A	Strong: Increase in percentage of participants who are extremely confident in their ability to assess a pregnant woman’s knowledge about breastfeeding (71 % at baseline and 84 % at endline)	Not asked, further investigation needed
17. Engage in a conversation with a pregnant woman about at least four care practices a mother/infant dyad will experience at the birthing facility that will support breastfeeding	Strong: Most know a reason for immediate and sustained mother–baby skin-to-skin contact after birth (100 % and 95 % at baseline and endline, respectively) and at least one factor that improves the mother’s childbirth experience (100 % at baseline and endline)	Strong: Improvements in the percentage of participants who were extremely confident in ability to explain the importance of skin-to-skin contact immediately after delivery from baseline to endline (76 % and 89 %, respectively) and to explain the importance of initiation of breastfeeding within 1 h after delivery (76 % and 89 %, respectively) Reinforcement needed: Decline in percentage of participants who were extremely confident in ability to describe how healthcare practices affect initiation of breastfeeding (76 % at baseline, 68 % at endline)	Not asked, further investigation needed
25. Engage in a conversation with a mother including at least three reasons why suckling at the breast in the first hour is important, when the baby is ready	Strong: Most know one reason that suckling at the breast within the first hour of birth is important at baseline and endline (81 % and 95 %, respectively)	Strong: Most participants are extremely confident in their ability to explain how breastfeeding works at baseline and endline (71 % and 74 %, respectively)	Not asked, further investigation needed
27. Describe to a mother at least three pre-feeding behaviours babies show before actively sucking at the breast.	Strong: Most know behaviours a baby should demonstrate instinctually before latching at baseline and endline (90 % and 95 %, respectively)	Reinforcement and further investigation needed: Just over two-thirds of participants were extremely confident in their ability to explain responsive feeding and its implications for the frequency and duration of breastfeeding (baby’s hunger and satiety cues) (71 % at baseline, 74 % at endline)	Not asked, further investigation needed
29. Engage in a conversation with a mother regarding at least three reasons why effective exclusive breastfeeding is important	Strong: Most know the global recommendation for duration of exclusive breastfeeding at baseline and endline (95 % and 100 %, respectively) and the importance of exclusive breastfeeding (90 % and 100 %, respectively)	Strong: Increase in percentage of participants who were extremely confident in their ability to explain the importance of exclusive breastfeeding for 6 months at baseline and endline (76 % and 89 %, respectively)	Not asked, further investigation needed
30. Engage in a conversation with a mother regarding two elements related to infant feeding patterns in the first 36 h of life	Strong: Most know what information to share with a mother about a newborn’s typical feeding patterns in the first 36 h of life at baseline and endline (100 % and 95 %, respectively)	Strong: Increase in percentage of participants who were extremely confident in their ability to counsel a pregnant woman about breastfeeding and infant feeding (67 % at baseline, 95 % at endline) Needs reinforcement: Just over two-thirds were extremely confident in their ability to explain infant feeding patterns in the first 36 h of life (76 % at baseline, 68 % at endline)	Not asked, further investigation needed
31. Describe to a mother at least four signs of adequate transfer of milk in the first few days	Strong: Most know a sign of adequate transfer of milk in the first few days at baseline and endline (90 % and 84 %, respectively)	Needs reinforcement: Three-quarters were extremely confident in their ability to list the signs and symptoms that indicate a newborn may not be getting enough milk (71 % at baseline, 79 % at endline) and explain the signs of adequate transfer of milk in the first few days (71 % at baseline, 74 % at endline)	Not asked, further investigation needed
32. Evaluate a full breastfeeding session, observing at least five points	Strong: Most know an important aspect that is observed at the end of a full breastfeeding assessment at baseline and endline (90 % and 89 %, respectively) and two things that should be observed when assessing a full breastfeeding session (95 % at baseline and endline)	Needs reinforcement: Three-quarters were extremely confident in their ability to assess a breastfeeding session using the appropriate job aid (76 % at baseline, 63 % at endline)	Not asked, further investigation needed
62. Develop individualised discharge feeding plans with a mother that includes at least six points	Strong: 100 % know the most important issue to discuss with a mother before she leaves the hospital after giving birth and when a mother should bring her baby to a healthcare professional after discharge at baseline and endline	Not asked, further investigation needed	Not asked, further investigation needed.
63. Describe to a mother at least four warning signs of infant undernourishment or dehydration for a mother to contact a healthcare professional after discharge	Reinforcement needed: Knowledge regarding warning signs of undernourishment or dehydration in the infant increased from 48 % at baseline to 53 % at endline	Needs reinforcement: Three-quarters were extremely confident in their ability to explain the warning signs of infant undernourishment or dehydration (71 % at baseline, 68 % at endline)	Not asked, further investigation needed
64. Describe at least three warning maternal signs for a mother to contact a healthcare professional after discharge	Not asked, further investigation needed	Needs further investigation, but most were extremely confident in their ability to counsel a mother about her own health (76 % at baseline, 84 % at endline)	Not asked, further investigation needed

Note: A successful mentorship programme for BFC is assured through knowledge, as the percent of the twenty-one knowledge questions answered correctly at baseline and endline confirms. The confidence levels to perform the thirty-two skills (measured by the thirty-two performance indicators) at baseline and endline stayed the same, indicating that the mentees have acquired and maintained the required attitude to provide quality BFC. This was verified using standardised detailed observation tools as shown in this table.

PNC, postnatal care, BFC, breastfeeding counselling.

#### Experiences with and perceptions of the mentorship programme

After the programme, all surveyed mentors and mentees said they would encourage others to join the programme (Table 4). The majority (89 %) of mentees indicated that the breastfeeding session observation job aid was the most helpful learning tool (Table 8 – see online supplementary material, Supplemental Material S2).

**Table 4.** Mentors’ and mentees’ perceptions of the BFC mentorship programme at endline

	Mentees		Mentors	
	*n* 19		*n* 7	
Perceptions of the BFC mentorship programme	*n*	%	*n*	%
Mentors felt very prepared to fill the role of mentor	N/A		6	86 %
Mentors felt the mentorship training was very helpful	N/A		7	100 %
Mentors understood what was expected of them as mentors	N/A		7	100 %
Mentors felt very satisfied with the support received from the facility for the mentorship programme	N/A		3	43 %
Mentors felt somewhat satisfied with the support received from the facility for the mentorship programme	N/A		4	57 %
Average time mentors spent on the mentorship programme per week				
1–4 h/week	N/A		3	43 %
5–8 h/week	N/A		1	14 %
> 8 h/week	N/A		3	43 %
Mentors felt this was too little time	N/A		4	57 %
Average time mentees spent with mentor per week				
< 3 h/week	7	37 %	N/A	
≥ 3 h/week	12	63 %	N/A	
Mentees’ perceptions of most important qualifications of mentors				
Experience	9	47 %	N/A	
Strong listening skills	18	95 %	N/A	
Strong counselling skills	17	89 %	N/A	
Knowledge of breastfeeding	16	84 %	N/A	
Supportive of mentees	17	89 %	N/A	
Respectful of mentees	15	79 %	N/A	
Mentees felt very respected by mentors	18	95 %	N/A	
Mentees felt that they met their BFC mentorship goals very well	17	89 %	N/A	
Would encourage others to join the BFC mentorship programme	19	100 %	7	100 %

BFC, breastfeeding counselling.

Most mentees (63 %) reported spending 4 or more hours per week with their mentor. Mentees felt that strong listening skills (95 %), counselling skills (89 %) and knowledge of breastfeeding (84 %) were important mentor qualifications, along with being supportive (89 %) and respectful (79 %) of mentees. Almost all mentees felt very respected by the mentors (95 %).

Six of the seven mentors felt very prepared for the BFC mentorship programme. All indicated that the mentor training was helpful, and they understood what was expected of them. All were satisfied with the support they received from health facility management – 43 % were very satisfied and 57 % were somewhat satisfied. The mentorship activities took a fair amount of time – 57 % of mentors reported spending 3–4 h per week per mentee. Mentors reported conducting an average of eight formal observations per mentee throughout the programme period, with most 63 % mentees spending 3 or more hours per week with their mentor.

### Quantitative findings from client exit interviews

#### Client characteristics

We interviewed 122 ANC clients (sixty-two at baseline, sixty at endline) and 121 PNC clients (sixty-one at baseline, sixty at endline) (Table 5). Most demographic characteristics were similar across time points for both groups. The median age of ANC clients was approximately 28 years, with most being married or cohabitating. For PNC clients, the percentage who were married or cohabitating increased from baseline to endline (88·5 % to 100 %, *P* = 0·01), and those with two or more previous births decreased (77 % to 55 %, *P* = 0·038). Other characteristics showed no statistically significant differences between time points.

**Table 5.** Demographic characteristics of ANC and PNC clients who received BFC at Mbagathi hospital

Socio-economic demographics	ANC clients (pregnant women)					PNC clients (postpartum women)				
	Baseline		Endline		*P*-value	Baseline		Endline		*P*-value
	(*n* 62)		(*n* 60)			(*n* 61)		(*n* 60)		
	*n*	%	*n*	%		*n*	%	*n*	%	
Median age in years	27·5		28·5		0·81	26·0		24·5		0·13
IQR	24·0–35·0		23·5–32·5			23·0–32·0		21·0–31·5		
Marital status										
Married or living with a partner	55	88·7 %	55	93·2 %	0·388	54	88·5 %	54	100·0	0·01
Single never married, divorced, or separated	7	11·3 %	4	6·8 %		7	11·5 %	0	0·0	
Level of education										
None	0	0 %	0	0 %	0·146	1	2 %	0	0 %	0·173
Primary	15	24 %	8	13 %		12	20 %	12	20 %	
Post-primary/vocational	0	0 %	0	0 %		10	16 %	6	20 %	
Secondary/A-level	32	52 %	28	47 %		24	39 %	35	58 %	
College/university	15	24 %	24	40 %		14	23 %	7	12 %	
Had paid work in the last 12 months	34	55 %	39	65 %	0·25	38	62 %	27	45 %	0·056
Number of previous births										
Median number of previous births	2·0		1·0		0·071	2·0		2·0		0·02
IQR	1·0–3·0		1·0–2·0			2·0–3·0		1·0–3·0		
None	8	13 %	7	12 %	0·112	N/A		N/A		0·038
1 birth	19	31 %	29	48 %		14	23 %	27	45 %	
2–6 births	35	56 %	24	39 %		47	77 %	33	55 %	
Number of ANC visits during this pregnancy										
Median number of ANC visits^††^	5·0		4·0		0·033			5·0		N/A
IQR	4·0–6·0		3·0–5·0					4·0–6·0		
1–3 visits	9	15 %	19	32 %	0·081	N/A		9	15 %	
4–7 visits	47	77 %	38	63 %		N/A		48	80 %	
8–10 visits	5	8 %	3	5 %		N/A		3	5 %	
Currently breastfeeding^†^						61	100 %	60	100 %	

The median number of births for the postpartum women at baseline was 2·0 (IQR 2·0–3·0) while at endline it was 2·0 (IQR 1·0–3·0). The IQR range at endline of 1·0–3·0 suggests that 50 % of women had between one and three births, showing a wider spread or greater variability in the number of births. This suggests that by the endline, there was a broader range of experiences regarding the number of births among the sampled population, with more women having fewer births (as low as one) compared with the baseline. ANC, antenatal care; PNC, postnatal care; BFC, breastfeeding counselling.

^†^PNC clients only; ^††^All clients excluding PNC pre-intervention.

#### Experiences and perceptions of breastfeeding counselling

Most clients interviewed reported being treated in a friendly manner by the health worker(s) providing BFC and support at baseline and endline (Table 6). Among ANC clients, there was no statistically significant change; however, among PNC clients, the percentage increased from 89 % to 100 % (*P* = 0·007). Similarly, ANC and PNC clients reported feeling respected by the health workers providing BFC both before and after the programme. ANC clients were more likely to have been addressed by name at endline (88 %) than at baseline (55 %) (*P* < 0·001).

**Table 6.** Pregnant and postpartum women’s perceptions of BFC, *n* (%)

	ANC clients (pregnant women)					PNC clients (postpartum women)				
	Baseline		Endline		*P* value	Baseline		Endline		*P* value
	(*n* 62)		(*n* 60)			(*n* 61)		(*n* 60)		
	*n*	%	*n*	%		*n*	%	*n*	%	
Client reported that the following happened ‘most’ and ‘all of the time’ during her visit, while receiving BFC										
Client was addressed by name during BFC	34	55 %	53	88 %	<0·001	22	36 %	29	48 %	0·17
Client was asked how she was feeling by health worker (HW) who provided BFC	52	84 %	54	90 %	0·43	51	84 %	53	88 %	0·59
Client felt she could ask HW who provided BFC any questions	55	89 %	53	88 %	0·95	52	85 %	56	93 %	0·15
Client felt treated in a friendly manner by HW who provided BFC	62	100 %	58	97 %	0·15	54	89 %	60	100 %	0·007
Client felt HW who provided BFC paid attention when they needed help with breastfeeding	N/A		N/A			58	95 %	58	97 %	0·66
Client felt respected by HW who provided BFC	61	98 %	59	98 %		58	95 %	60	100 %	0·082
Client was asked for consent before breastfeeding observation/help during BFC	N/A		N/A			46	75 %	34	57 %	0·029
Client felt she could discuss problems with HW who provided BFC without others not involved in care overhearing (privacy)	N/A		N/A			27	44 %	42	70 %	0·004
Client felt BFC was helpful	59	95 %	58	97 %	0·56	61	100 %	60	100 %	N/A
Client felt BFC gave her knowledge about breastfeeding	45	73 %	58	97 %	<0·001	N/A		N/A		N/A
Client reported that the following ‘never’ happened during her visit while receiving BFC										
Client felt discriminated based on personal attributes during BFC	1	2 %	1	2 %	0·98	7	11 %	1	2 %	0·03
Client felt physically mistreated during BFC	0	0 %	0	0 %	N/A	2	3 %	0	0 %	0·16
Client felt verbally mistreated during BFC	3	5 %	1	2 %	0·33	4	7 %	0	0 %	0·044

Notes: We measured health worker practices during BFC, reported during client exit interviews using a Likert scale (no, never = 0; yes, some of the time = 1; yes, most of the time = 2; and yes, all of the time = 3) and converted into a binary outcome of either 1 = yes, all or most of time for positive statements or 1 = any yes for negative statements to simplify and reduce the response categories presented here. cccc PNC, postnatal care; BFC, breastfeeding counselling.

At baseline, only 3 % of PNC clients interviewed reported physical mistreatment by the health worker(s) providing BFC and support. None of the ANC or PNC clients reported physical mistreatment at endline. Among PNC clients, the percentage who reported verbal mistreatment declined (7 % baseline; 0 % endline, *P* = 0·04) as did the percentage who felt discriminated against based on personal attributes (from 11 % to 2 %, *P* = 0·03). More PNC clients indicated that they had privacy during BFC at endline (70 %) than at baseline (44 %) (*P* = 0·004). The percentage of PNC clients who reported health workers asking for consent before observing/helping with breastfeeding declined from 75 % at baseline to 57 % at endline (*P* = 0·029). Both ANC and PNC clients indicated that BFC was useful, and the percentage of ANC clients who felt that BFC gave them more knowledge increased from 73 % at baseline to 97 % at endline (*P* < 0·001).

#### Qualitative findings from focus group discussion and key informant interview

We identified several themes from the qualitative data collected through the FGD and KII. Key informants indicated that the mentorship programme was integrated with existing implementation of national guidelines on quality obstetrics and perinatal care^(30)^ at the hospital, routine reporting, continuous medical education and continuous quality improvement. For example, strengthening monthly reporting on early initiation of breastfeeding by implementing skin-to-skin contact immediately after delivery aligns with mentorship programme goals to improve the quality of BFC and breastfeeding outcomes and immediate care of the newborn, a key action in the national guidelines.

Mentors and mentees were interested in participating to increase their knowledge and practices related to breastfeeding and improve maternal and child health. Mentors and mentees felt that gaining confidence to counsel caregivers on breastfeeding was one of the main positive outcomes. Health workers also mentioned strengthening foundational counselling skills, such as listening to and learning from the client. They perceived improvements in breastfeeding practices as well as maternal and child health outcomes and attributed them to the programme.

*‘It has changed me. As I carry out my daily duties, I can assist a mother whose child is not breastfeeding well. The program has given me the ability to help such mothers, not only in the workplace but also outside my workplace. I can confidently implement the BFHI.’ (ANC Mentees FGD, health worker [HW] 2)*

Mentees felt that mentors were well-qualified and could influence health workers.

*‘[My mentor] was quite knowledgeable on what should be done. So whenever we faced some challenges, especially on the filling of the books and some scales, she could clarify how much is needed.’ (PNC Mentees FGD, HW 5)*

They thought it was strategic to select departmental heads to serve as mentors.

*‘Mentors were primarily chosen from among those who were already in charge of departments … this method was seen as strategic because it utilized existing hierarchies.’ (ANC Mentees FGD, HW 1)*

Mentees generally viewed the selection and matching process positively, particularly the strategic use of existing leadership structures, merit-based selection and observations made during the trainings.

*‘I think the process was strategic because by starting with the departmental heads those who were interacting with ANC and PNC, it gives them the leeway to spearhead the process and number two, when it comes to selecting the mentees the people working under them, I think it went well it is like trickling down effect down the chain.’ (ANC Mentees FGD, HW 2)*

However, mentees noted that mentors’ other responsibilities made it hard to find time for mentoring and caused interruptions. Both mentors and mentees mentioned challenges scheduling meetings for debriefing, providing feedback or discussing issues. They noted that this was particularly challenging when mentors and mentees worked in different departments or had different work schedules.

*‘A recurring issue was the lack of sufficient staff to handle the high volume of deliveries and have adequate time to provide breastfeeding counselling.’ (ANC Mentees FGD, HW 2)*

*‘My mentor is a nutritionist and I’m a nurse. The days that we’re supposed to meet, we find that we are in different shifts.’ (PNC Mentees FGD, HW 7)*

Mentors and mentees were able to overcome these challenges by creating WhatsApp groups. The WhatsApp group served as a platform for ongoing support between face-to-face mentorship sessions, especially when scheduling conflicts arose due to different work shifts or departments. Mentees particularly valued the ability to receive real-time advice on complex cases through shared photos and descriptions. As one mentee noted.

*‘For me I can say, it somehow went well. Despite the fact that we are different cadres, we were able to create a WhatsApp where we could do our meetings and communicate.’ (PNC Mentees FGD, HW 6)*

Mentees appreciated the range of approaches used by mentors. Mentors expressed appreciation for the job aids guiding them, particularly when observing BFC sessions. Both mentees and mentors commented on the supportive approach that mentors took to providing feedback to mentees.

*‘It was also helpful in that she would actually appreciate what you have done good, and later she would come up with what you could have done better. That encouraged us, gave us encouragement.’ (PNC Mentees FGD, HW 4)*

Finally, mentees noted that the mentorship programme fostered relationships and teamwork across different cadres.

*‘Teamwork, especially among health workers, nutritionists, nurses, and the administration, has really helped in the implementation of the program because if it was one cadre doing everything, it would not be successful as it is now. Because most of the cadres, we are now working together well.’ (PNC Mentees FGD, HW 1)*

#### Factors that enabled and hindered implementation

Several factors enabled the implementation of the programme (Table 7). First and importantly, we designed the programme to be an integral part of health facility activities and implemented by staff. Second, health facility management had ownership of, support for and commitment to the study intervention. Leadership support for proposed changes in service delivery practices has been shown to be critical for engaging staff at all levels^(31)^. Third, conducting the BFHI training prior to the programme helped to ensure that mentees had the foundational knowledge of BFC prior to the programme. Fourth, mentors and mentees indicated they wanted to improve their knowledge and skills, women’s breastfeeding knowledge and practices and, ultimately, maternal and child health outcomes. Wallen and colleagues showed that believing in the importance of new practices contributes to their use. Mentors and mentees perceived improvements and attributed them to the programme, which seems to have motivated them to actively participate for the duration^(31)^. Fifth, mentors and mentees noted that mentors were well matched with mentees with whom they had a good rapport, who worked in the same department and/or were of the same cadre, thus ensuring compatibility, which is critical for successful implementation. Sixth, selecting trusted, well-respected and influential individuals as mentors and matching them with mentees with similar work schedules facilitated the implementation of the intervention and helped mentees be open to receiving feedback and suggestions for improvement. Seventh, mentors commented on the usefulness of the job aids, particularly the observation tools, which focussed on specific competencies related to mentees’ actions and skills and counselling checklists. Finally, mentors appreciated the programme’s versatility. Choosing the mentorship approach(es) that worked best for them and their mentees (i.e. in-person group, one-on-one, peer-to-peer or virtual meetings; demonstrations; observations; debriefs; teaching sessions and discussions) empowered mentors.

**Table 7.** Enablers and barriers of the facility-based mentorship programme for BFC

Enablers	Description
Merit-based selection	Facility-based mentors were chosen from senior experienced staff, leveraging existing authority and respect to facilitate effective mentorship
Training for mentors	Mentors received training on core principles of mentorship before assuming their roles, equipping them with the necessary skills and knowledge
Multidisciplinary engagement	The programme encouraged collaboration among various cadres of healthcare professionals, enhancing support for BFC
Integration with hospital programmes	The mentorship was integrated into existing hospital programmes, such as monthly continuing medical education sessions, quality improvement and reporting activities for key national indicators
Structured implementation	The facility-based mentoring processes were well-structured and adapted to fit within normal hospital client care activities
Barriers	Description
Structural limitations	Initially, there were insufficient delivery beds, which hindered skin-to-skin contact and initiation of breastfeeding immediately after birth, and little privacy during BFC in the postnatal ward
Staffing shortages	A lack of sufficient staff to handle the high volume of deliveries in the labour ward and subsequently provide adequate support for BFC was a recurring issue
Training coverage for all department staff	Not all staff were selected for the maternity course training, leading to inconsistencies in the care practices and BFC support provided to mothers
Misinformation and poor community engagement	Mothers often started ANC in their last trimester, but some had their first contact during delivery at the facility. These mothers arrived with incorrect information about breastfeeding, requiring health workers to spend much more time counselling
Scheduling conflicts	Organising meetings was challenging due to scheduling conflicts for those whose mentors were only available during day shifts, while they were scheduled for night shifts
Lack of clarity in the selection criteria for mentors	Some mentees were unclear about the criteria used for selecting mentors, leading to perceptions of bias, since most of the mentors were experienced senior staff
Over-reliance on hierarchical positions in departments as mentors	Using senior staff as mentors who had competing tasks and limited time might limit mentoring and learning opportunities
Commitment and personal sacrifice	Participation in the programme required a significant time commitment, often outside of regular working hours

BFC, breastfeeding counselling; ANC, antenatal care.

Nonetheless, there were challenges due to heavy workloads and conflicting work schedules. The former may be less of an issue in lower-level facilities not receiving a high number of referrals. The latter was mostly an issue when mentors were paired with mentees from different cadres.

## Discussion

Mentorship programmes can improve the quality of maternal, neonatal and child healthcare services, including in resource-limited settings^(22–24,32)^. Mentorship has also been found to influence mentee attitudes, interpersonal relations and motivation^(25,31,33)^.

This feasibility study expands the evidence base by focusing on the feasibility of a mentorship programme for BFC. We determined whether the mentorship programme improved mentees’ knowledge and confidence to provide quality counselling and pregnant and postpartum women’s perceptions of BFC.

### Mentees’ knowledge and confidence related to breastfeeding counselling

While mentees demonstrated high baseline knowledge (94 % correct) and confidence, which limited the potential for significant improvements across all indicators, it is noteworthy that significant change was observed specifically in mentees’ confidence in counselling women on breastfeeding and infant feeding (from 67 % at baseline to 95 % at endline, *P* = 0·014). The lack of statistically significant changes in other knowledge and confidence indicators should be interpreted in the context of already high baseline scores and the small sample size (*n* 21 at baseline, *n* 19 at endline). Rather than suggesting the programme had limited impact on knowledge^(34–36)^, these findings indicate that the mentorship approach may be most valuable for enhancing the capability to use a set of related knowledge, skills and behaviours to successfully provide BFC in a clinical setting.

### Pregnant and postpartum women’s perceptions of breastfeeding counselling

Nearly all clients interviewed reported health workers treating them in a friendly manner and feeling respected before and after the mentorship programme. They did not report feeling discriminated against or experiencing physical or verbal mistreatment. Notably, we observed a significant decline among PNC clients who felt verbally mistreated and discriminated against based on personal attributes. This improvement in client treatment aligns with the FGD; mentees indicated increased empathy for clients. Finally, ANC and PNC clients’ perception of the usefulness of BFC increased over time.

A somewhat surprising finding was the decline in the percentage of PNC clients who reported health workers asking for consent before observing or helping with breastfeeding. This might have to do with (a) BFC becoming so integrated into services that health workers overlooked the need to ask each time and/or (b) enthusiasm to help women breastfeed might have led to health workers forgetting to ask for consent.

### Study limitations

The study was conducted at a single health facility, limiting generalisability to other levels of care. Without a comparison group and purposive/convenient sampling, changes in mentee confidence and client-reported counselling cannot be definitively attributed to the mentorship programme. Our sample sizes were not large enough to detect moderate differences between baseline and endline. Exit interviews focused on satisfaction and measures of respective care rather than practices related to prioritised competencies. As a repeat cross-sectional design, there were some statistically significant differences between ANC and PNC client groups at baseline and endline. Overall, while suggestive, the lack of a comparison group limits definitive conclusions about the programme’s impact. A limitation of this feasibility study was the lack of data on breastfeeding outcomes such as initiation rates and exclusive breastfeeding rates at hospital discharge. Future evaluations of the facility-based BFC programme should include these metrics to assess the ultimate impact on breastfeeding practices.

### Conclusions

The BFC mentorship programme was feasible when implemented as designed in a health facility with the requisite capacity to adapt it based on its existing infrastructure and supportive leadership.

As a next step, we recommend a series of carefully designed pilot studies implemented in a variety of settings (urban, rural), socio-economic environments and health facility types (public and private facilities, primary and secondary care facilities). These pilot studies will guide the next phase of implementation and enhance already existing efforts by respective county health departments implementing BFHI to achieve Step 2. Additionally, further research should assess the impact of the mentorship programme on breastfeeding practices among mothers in Kenya. This approach will provide valuable insights into the scalability and adaptability of the mentorship programme in different contexts and will ultimately contribute to improved BFC and breastfeeding outcomes.

Facility-based mentoring to strengthen BFC competencies is one potential approach for countries to achieve the second step of the BFHI ten steps. Based on study findings, implementation guidance^(26)^ was revised to include: (1) updated BFHI competencies; (2) evidence of adaptability across facility levels; (3) enhanced mentor–mentee pairing and support mechanisms, including virtual options and (4) simplified competency assessment tools linked to various service points These evidence-informed refinements enhance the programme’s contextual adaptability whilst preserving fidelity to its foundational components, thus facilitating wider implementation across diverse healthcare settings.

## Supporting information

Njoroge et al. supplementary material Njoroge et al. supplementary material

## Supplementary material

For supplementary material accompanying this paper, visit https://doi.org/10.1017/S1368980025100591
