# Supplementary material for: Breastfeeding counselling mentorship programme feasibility: a mixed-methods study
Source: Public Health Nutr. 2025 Jul 4;28(1):e200. doi: 10.1017/S1368980025100591 (PMC12951348; doi:10.1017/S1368980025100591)
Supplement: Njoroge et al. supplementary material [file S1368980025100591sup001.docx]

### Supplementary Materials

#### S1 Sampling Plan

##### Sample Size Calculation for Client Exit Interviews

The sample size for client exit interviews was calculated based on an estimated 18 live births per day, resulting in approximately 252 live births over the 2-week data collection period at each time point. To achieve a 90% confidence level with a 10% margin of error, a sample size of 52 per time point per unit/clinic was required. Accounting for a 10% nonresponse rate, the target sample size was adjusted to 60 per time point per unit/clinic.

The sample size (*n*) and margin of error (*E*) are given by:

$$n=\frac{N-x}{\left( \left( N-1 \right)E^{2} \right)+x}$$

$$x=Z\left( \frac{c}{100} \right)^{2r\left( 100-r \right)}$$

$$E=\left[ \sqrt{\left( N-n \right)x}/n\left( N-1 \right) \right]$$

where *N* is the population size, *r* is the fraction of responses that you are interested in, and *Z*(*c*/100) is the critical value for the confidence level *c*. The Raosoft sample size calculator was used for these calculations.

#### S2 Summary of Responses from Mentees on the Most Helpful Materials

Table 8. Summary of Responses from Mentees on the Most Helpful Materials

| Materials | **Mentees**  **N= 19**  **N (%)** |
| --- | --- |
| Observation Tool 1: Antenatal Care Services | 8 (44%) |
| Observation Tool 2: Postnatal Care Unit | 9 (50%) |
| Job Aid: Breastfeeding Session Observation Checklist | 16 (89%) |
| Job Aid: Antenatal Checklist―Infant Feeding | 8 (44%) |
| Examiner’s Resource | 2 (11%) |
| Checklist: Topics to Cover During Counseling Visits in Antenatal Care Services | 8 (44%) |
| Checklist: Topics to Cover During Counseling Visits in the Postnatal Care Unit | 10 (53%) |

#### S3 Minimum Requirements for Selecting Mentors

The following are the minimum requirements used for selecting a facility mentor:

1. Completed the BFHI training (maternity course)
2. Provides direct care to clients at one of the prioritized service delivery point(s)
3. Has met all qualifications to work as a doctor, nurse, nutritionist, clinical officer, or midwife
4. Has a minimum of 2 years of experience providing maternal and newborn care, but 5 or more years is preferred
5. Demonstrates a high level of competency (i.e., knowledge, skills, and attitudes) in providing breastfeeding counselling (BFC), observing BFC, and accurately assessing BFC competencies
6. Senior management is supportive and agrees to allow staff to serve as a mentor
7. Conversant with the Breast Milk Substitutes (BMS) Act of 2012, the International Code of Marketing of BMS, and the subsidiary BMS regulations of 2021
8. Avoids conflicts of interest particularly with companies that produce BMS designated products, their parent or subsidiary companies, and political leaders. This is imperative to ensure direct care providers protect families from commercial pressure. All mentors must sign a code of conduct indicating adherence to this.

##### Additional Considerations for Selecting Mentors

The BFHI Implementation Team can also guide the selection of mentors based on the understanding of the workplace culture, size of the facility, number of facility service delivery points chosen, number of possible mentees, and individual experience. For selection of the most effective mentors, in addition to considering the minimum requirements, the BFHI facility implementation team may also wish to consider the following:

1. Availability
2. Other responsibilities
3. Reliability
4. Level of professional expertise
5. Experience being in a supervisory role
6. Experience with mentoring and pre-service professional education
7. Ability to be a leader and influence people
8. Knowledge about maternity care and infant feeding practices within the BFHI context to accurately detect both correct and incorrect knowledge, skills, and attitudes (behaviors)
9. Communication and organizational skills
10. Critical thinking and problem-solving skills
11. Resilience and adaptability
12. Supportive and positive attitude in approach to work
13. Attention to detail
14. Commitment to improving the quality of care for patients
15. Willingness to learn new skills
16. Willingness to commit to participating in the mentorship activities for the duration of the mentorship program.

#### S4 Minimum Requirements Used for Selecting Mentees

The implementation guidance indicates that the BFHI facility implementation team, in consultation with mentors, selects mentees according to a set of criteria and the minimum requirements, using findings from the facility self-assessment and complementary assessment activities.

Mentees are direct care providers who deliver BFC to clients. They are dedicated, skilled health providers who seek to grow and develop personally and professionally to successfully achieve their goals and strengthen their BFC competencies with the support of a mentor.

The following are the minimum requirements for mentees:

1. Completed the BFHI training
2. Provides direct care to clients at one of the prioritized service delivery point(s)
3. Has met all qualifications to work as a doctor, nurse, nutritionist, clinical officer, midwife, or breastfeeding peer supporter
4. Senior management is supportive and agrees to allow staff to participate as a mentee
5. Avoids conflicts of interest particularly with companies that produce BMS designated products, their parent or subsidiary companies, and political leaders. This is imperative to ensure direct care providers protect families from commercial pressure. All mentees must sign a code of conduct indicating adherence to this.

##### Additional Considerations for Selecting Mentees

In addition to considering the workplace environment, number of mentors available, and service delivery point(s) prioritized for the program, the BFHI facility implementation team may also wish to consider the following factors when selecting a mentee:

1. Availability
2. Other responsibilities
3. Reliability
4. Willingness to learn new skills
5. Positive attitude in approach to work
6. Commitment to improving the quality of care for patients
7. Open to receiving feedback and guidance from their mentor
8. Willingness to commit to participating in the mentorship activities for the duration of the mentorship program.

#### S5 Health Worker Post-training Survey

This survey or test was developed by the United States Agency for International Development (USAID) Advancing Nutrition project, the Kenya Ministry of Health (MoH), Division of Nutrition and Dietetics, and Mbagathi County Referral Hospital.

It was designed as an online health worker self-administered survey tool formatted for use on mobile phones. The tool assessed changes in health workers’ knowledge and confidence to conduct tasks related to the priority BFC competencies.

1. Have you completed a consent form?

- Yes
- No → Please inquire with the study team and complete an informed consent form before continuing.

1. Unique ID ___ ___

**Knowledge related to breastfeeding counseling**

First, we would like to see what you learned from the BFHI training. This is not to test you, but to assess the training.

1. Which is an open-ended question? (select one response)

- Is there someone who supports your feeding decisions?
- **What have you heard about breastfeeding?**
- Are you planning to breastfeed?
- Did your mother breastfeed?
- Don’t know / not sure

1. Which is an open-ended question? (select one response)

- Did you take a breastfeeding class?
- Are you planning to breastfeed?
- **What are your plans and goals for feeding your baby?**
- Did your mother or grandmother breastfeed?
- Don’t know / not sure

1. What is the global recommendation for duration of exclusive breastfeeding? (select one response)

- At least one month
- Four to six months
- Twelve months
- **Six months**
- Don’t know / not sure

1. What is the global recommendation for how long a baby should be breastfed? (select one response)

- Until the mother’s milk dries up
- **Two years or longer**
- As long as possible
- At least 1 year
- Don’t know / not sure

1. What is the global recommendation for when breastfeeding should begin? (select one response)

- By 24 hours after birth
- Within 12 hours after birth
- **Immediately after birth (within the first hour)**
- When the mother’s milk comes in
- Don’t know / not sure

1. Which of the following is a risk for babies who are not breastfed? (select one response)

- **Higher risk of diarrhea**
- Higher risk of not bonding with father
- Higher risk of guinea worm infection
- Higher risk of malaria
- Don’t know / not sure

1. Which of these is NOT a reason why breastfeeding is important for the baby? (select one response)

- It provides long-term protection against chronic non-communicable diseases. Breastfed babies remain closer to their mothers throughout their lives.
- It is easily digested and efficiently used by the baby’s body. Formula feeding allows the baby’s father to be more involved.
- It protects a baby against infection. I do not believe it is different from formula feeding.
- **It cools the baby down when hot.**
- Don’t know / not sure

1. Why is breastfeeding important for the mother? (select one response)

- It is part of a mother's duty.
- **It reduces the risk of breast and ovarian cancer.**
- It reduces the risk of high cholesterol.
- It allows her to get pregnant soon after giving birth.
- Don’t know / not sure

1. Which is an important reason for immediate and sustained mother-baby skin-to-skin contact after birth? (select one response)

- **The baby is colonized with the mother's normal and healthy bacteria, and the baby is warmed by the mother's body.**
- The healthcare provider can do all the post-birth procedures.
- Mother is colonized with baby's bacteria.
- Mother can tell the gender of the baby.
- Don’t know / not sure

1. Which of these topics would be your priority when discussing breastfeeding with a pregnant woman? (select one response)

- Special foods that will help her make more milk.
- **Early and exclusive breastfeeding.**
- Introduction of complementary foods.
- What kind of feeding bottles are best.
- Don’t know / not sure

1. What will you make sure to discuss with a pregnant woman about breastfeeding? (select one response)

- **The importance of breastfeeding and exclusivity.**
- The importance of making sure of the partner's approval.
- The importance of her mother's approval.
- The importance of preparing her breasts for breastfeeding.
- Don’t know / not sure

1. Name at least one factor that improves the mother's childbirth experience. (select one response)

- Routine shaving of pubic hair and administering an enema.
- **Emotional support from the continuous presence of a companion of choice.**
- Requiring mother to lie flat on her back during labor.
- Withholding food and fluid during labor.
- Don’t know / not sure

1. What is one reason that suckling at the breast within the first hour of birth is important? (select one response)

- Prevents infant dehydration.
- Normalizes baby’s blood sugar (glycemia).
- Allows mother to safely rest.
- **Triggers onset of milk production.**
- Don’t know / not sure

1. When a baby is placed skin-to-skin on the mother at birth, what behaviors should they demonstrate instinctually before latching? (select one response)

- Slowly calming down so a helper can assist the baby to reach the breast
- Crying vigorously and then resting without movement.
- **Moving to the breast and touching the mother's body and breast.**
- Slowly going into deep sleep then starting to move hands and feet.
- Don’t know / not sure

1. Which statement about exclusive breastfeeding is correct? (select one response)

- Mothers do not have enough breast milk in the first few days.
- Some supplementation with artificial milk won't alter the intestinal microflora.
- **Baby will get all the nutrients needed by effective exclusive breastfeeding.**
- Mother needs to exclusively breastfeed every 3–4 hours to have enough milk.
- Don’t know / not sure

1. What information would you share with a mother about a newborn's typical feeding patterns in the first 36 hours of life? (select one response)

- Feeding patterns are determined by the mother so that the infant is correctly trained to a feeding schedule.
- Mother should only feed the baby six times per 24 hours.
- **Minimum feeding frequency is eight times per 24 hours.**
- Cluster feeding indicates low milk transfer and baby necessitates supplementation.
- Don’t know / not sure

1. Which of the following is a sign of adequate transfer of milk in the first few days? (select one response)

- Stools are dark for the first week of life.
- At least four stools by day 2.
- Baby has a large stool every day.
- **Baby passes meconium stool followed by increase in stool output.**
- Don’t know / not sure

1. Which of these should be observed during a full breastfeeding assessment? (select one response)

- **Mother’s breasts and nipples are intact and comfortable (absence of breast or nipple pain).**
- Baby brings fist to mouth and begins sucking again.
- Mother’s nipple is creased at the tip.
- Milk is spurting from the mother’s breast and the breast is still feeling full.
- Don’t know / not sure

1. What are two things that should be observed when assessing a full breastfeeding session? (select one response)

- **Infant has rhythmic bursts of sucking with brief pauses; the infant releases the breast at the end of feed in obvious satiation.**
- Mother's nipples hurt a little at the beginning of the feed; the infant has rhythmic bursts of sucking.
- Mother supports the infant's head; mother admits her nipples hurt a little during feeds.
- Infant has sucking movements at the jaw; the infant sucks at both breasts.
- Don’t know / not sure

1. What is the most important issue to discuss with a mother before she leaves the hospital after giving birth? (select one response)

- **What it means to exclusively breastfeed.**
- How to correctly use her breast pump.
- What kind of nipple cream to get for sore or cracked nipples.
- When to start feeding her baby meat.
- Don’t know/not sure

1. What information would you share with a mother about when she should bring her baby to a healthcare professional after discharge? (select one response)

- Baby has yellowish stool more than three times a day.
- Baby feeds eight or more times per 24 hours.
- Baby sleeps less than 4 hours at a time.
- **Baby has a scant amount of urine per day.**
- Don’t know / not sure

1. Which of the following is a warning sign of undernourishment or dehydration in the infant? (select one response)

- Stools are mustard-colored and the consistency of yogurt.
- Most feeds last only 20 minutes.
- Baby swallows after every 3–4 sucks.
- **Baby is regularly sleeping for more than 4 hours at a time in the first week and is difficult to arouse.**
- Don’t know / not sure

Before moving on to our final set of questions, please check your answers. You will not be able to revise them after moving on to the next question.

**Perceptions related to the breastfeeding counseling program**

1. How helpful did you think the BFHI training was for preparing you to provide quality breastfeeding counseling? (select one response)

- Very helpful
- Somewhat helpful
- Neutral
- Somewhat unhelpful
- Very unhelpful

**Confidence with breastfeeding counseling**

Next, we would like to know how confident or certain you are in your ability to conduct various activities that may be required when providing breastfeeding counseling.

1. Overall, how confident or certain are you in your ability to provide quality breastfeeding counseling? (select one response)

- Not at all confident
- Slightly confident
- Somewhat confident
- Quite confident
- Extremely confident

1. Finally, I would like to know how confident or certain you are in your ability to conduct various activities that may be required when providing breastfeeding counseling.

| **How confident are you in your ability to… (select one response for each activity)** | **Not at all confident** | **Slightly confident** | **Somewhat confident** | **Quite confident** | **Extremely confident** | **Don’t know/not sure** |
| --- | --- | --- | --- | --- | --- | --- |
| 1. **Use** the listening and learning skills to counsel a mother/parent/caregiver |  |  |  |  |  |  |
| 1. **Use** the skills for building confidence and giving support to counsel a mother/caregiver |  |  |  |  |  |  |
| 1. **Assess** a pregnant woman’s knowledge about breastfeeding |  |  |  |  |  |  |
| 1. **Assess** a breastfeeding session using the Job Aid: Breastfeeding Session Observation |  |  |  |  |  |  |
| 1. **Help** a mother to position her baby for breastfeeding using the four key points of positioning (baby’s head and body in line; baby held close to mother’s body; baby’s whole body supported; baby approaches breast, nose to nipple) |  |  |  |  |  |  |
| 1. **Explain** to a mother the four key points of attachment for breastfeeding (Is there more areola above the baby’s top lip than below? Is the baby’s mouth open wide? Is the lower lip turned outward? Does the baby’s chin touch the breast?) |  |  |  |  |  |  |
| 1. **Help** a mother to attach her baby to the breast once they are well positioned |  |  |  |  |  |  |
| 1. **Explain** to a mother the importance of exclusive breastfeeding for 6 months |  |  |  |  |  |  |
| 1. **Explain** to a mother the importance of continued breastfeeding for up to 2 years and beyond |  |  |  |  |  |  |
| 1. **Explain** to a mother about responsive feeding and its implications for the frequency and duration of breastfeeding (being sensitive to their baby’s hunger and satiety cues) |  |  |  |  |  |  |
| 1. **Explain** to a mother the importance of skin-to-skin contact immediately after delivery |  |  |  |  |  |  |
| 1. **Explain** to a mother the benefits of breastfeeding to the baby |  |  |  |  |  |  |
| 1. **Explain** to a mother the steps of expressing breast milk by hand |  |  |  |  |  |  |
| 1. **Practice** with a mother how to cup feed her baby safely |  |  |  |  |  |  |
| 1. **Counsel** a pregnant woman about breastfeeding and infant feeding |  |  |  |  |  |  |
| 1. **Explain** the importance of skin-to-skin contact immediately after delivery and the initiation of breastfeeding within 1 hour |  |  |  |  |  |  |
| 1. **Explain** the importance of initiation of breastfeeding within 1 hour after delivery |  |  |  |  |  |  |
| 1. **Describe** how healthcare practices affect initiation of breastfeeding |  |  |  |  |  |  |
| 1. **Explain** to a mother the benefits of breastfeeding to the mother |  |  |  |  |  |  |
| 1. **Explain** to a mother the benefits of breastfeeding to the child |  |  |  |  |  |  |
| 1. **Explain** to a mother how breastfeeding works |  |  |  |  |  |  |
| 1. **Explain** to a mother infant feeding patterns in the first 36 hours of life |  |  |  |  |  |  |
| 1. **List** the signs and symptoms that indicate a newborn may not be getting enough milk |  |  |  |  |  |  |
| 1. **Explain** to a mother the signs of adequate transfer of milk in the first few days |  |  |  |  |  |  |
| 1. **Explain** to a mother the warning signs of infant undernourishment or dehydration |  |  |  |  |  |  |
| 1. **Recognize** breast refusal and help a mother to breastfeed |  |  |  |  |  |  |
| 1. **List** the different reasons why a newborn may cry often |  |  |  |  |  |  |
| 1. **Help** a mother who has flat or inverted nipples |  |  |  |  |  |  |
| 1. **Help** a mother with engorged breasts |  |  |  |  |  |  |
| 1. **Help** a mother with sore or cracked nipples |  |  |  |  |  |  |
| 1. **Help** a mother with mastitis |  |  |  |  |  |  |
| 1. **Describe** alternative methods of feeding |  |  |  |  |  |  |
| 1. **Counsel** a mother about her own health |  |  |  |  |  |  |
| 1. **Implement** the International Code of Marketing of Breast-Milk Substitutes in a health facility |  |  |  |  |  |  |

31. Is there anything else you would like to mention about the BFHI training or breastfeeding counseling? Thank you so much for your time!

| **QUESTION** | **CODING CLASSIFICATION** | **SKIP** |
| --- | --- | --- |
| As a reminder, if, at any time, you do not want to answer a question or discuss an issue, you are free to decline to do so. You are also free to stop the interview at any time. The decision about whether or not to answer any specific question will not affect the services you receive at any health facility today or any time in the future.  *Ikiwa, wakati wowote, hutaki kujibu swali au kujadili suala, uko huru kukataa kufanya hivyo. Uko huru pia kujiondoa kwenye utafiti wakati wowote. Uamuzi kuhusu kushiriki au kutoshiriki katika utafiti huu au kujibu swali lolote maalumu hautaathiri haki zako za kupokea huduma zozote katika vituo vyovyote vya afya leo au wakati wowote ujao.* | | |
| 1. First, what is your new baby’s name? I will not write this down. I just want to be able to refer to her or him by name.   *Jina la mtoto aliyejifungua ni lipi? sitaandika jina lake nataka tu kurejelea kwa jina.* | DO NOT RECORD THE NAME.  NO NAME YET 1  PREFER NOT TO ANSWER 9 | . |
| 1. Next, how old were you at your last birthday? *Ulikuwa na umri gani katika siku yako ya kuzaliwa ya mwisho.* | ___ ___ years  DK 888 |  |
| 1. Have you ever been married? Are you currently married, living with a partner as if married, widowed, divorced, or separated? [DO NOT READ RESPONSE OPTIONS.]   *Je, kwa sasa umeolewa, unaishi na mwenzi wako kana kwamba umeolewa, umefiwa na mumewe/Mjane, umetalikiana, au umetengana?* [DO NOT READ RESPONSE OPTIONS.] | CURRENTLY MARRIED 2  LIVING WITH A PARTNER AS IF MARRIED 3  WIDOWED 4  DIVORCED OR SEPARATED 5  PREFER NOT TO ANSWER 9 |  |
| 1. Have you ever attended school? [DO NOT READ RESPONSE OPTIONS.]   *Uliwaahi kwenda shule kusoma?* [DO NOT READ RESPONSE OPTIONS.] | YES 1  NO 2 →  PREFER NOT TO ANSWER 9 → | GO TO Q6  GO TO Q6 |
| 1. What is the highest level of school you attended: primary, post-primary/vocational, secondary, college (middle level), university or higher?   *Ni kiwango gani cha juu zaidi cha shule ulichosoma: Shule ya msingi, baada ya shule ya msingi au ya ufundi, sekondari, chuo (kiwango cha kati), chuo kikuu au zaidi?* | PRIMARY 1  POST-PRIMARY/ VOCATIONAL 2  SECONDARY 3  COLLEGE (MIDDLE LEVEL) 4  UNIVERSITY OR HIGHER 5  PREFER NOT TO ANSWER 9 |  |
| 1. Aside from your own housework, have you done any work in the last 12 months? This includes a job for which you were paid in cash or kind. It includes small businesses or work on the family farm or in the family business. [DO NOT READ RESPONSE OPTIONS.] *Kando ya kazi zako za nyumba, uliwahi kufanya kazi ingine kwa kipindi cha miezi 12 iliyopita?* [DO NOT READ RESPONSE OPTIONS.] | YES 1  NO 2 →  PREFER NOT TO ANSWER 9 → | GO TO Q9  GO TO Q9 |
| 1. Are you paid in cash or kind? [DO NOT READ RESPONSE OPTIONS.] *Je, unalipwa pesa au kwa njia nyingine?*  [DO NOT READ RESPONSE OPTIONS.] | CASH ONLY 1  CASH AND KIND 2  IN KIND ONLY 3  NOT PAID 4  PREFER NOT TO ANSWER 9 |  |
| 1. What is your occupation? By this I mean, what kind of work do you mainly do? [RECORD HER RESPONSE IN THE SPACE PROVIDED.] *Kazi yako ni nini? Kwa hili namaanisha, ni aina gani ya kazi unayofanya hasa?*  [RECORD HER RESPONSE IN THE SPACE PROVIDED.] |  |  |
| 1. Finally, how many children have you given birth to?   *Mwishowe, umejifungua watoto wangapi?* NOTE: THIS INCLUDES LIVING OR DEAD, LIVING WITH THEM OR AWAY FROM HOME. | # OF CHILDREN: ___ ___  DK 88  PREFER NOT TO ANSWER 99 |  |
| Now, I am going to ask you some additional questions about specific aspects of the breastfeeding counseling and support you received from health facility staff at Mbagathi after giving birth to [BABY'S NAME]. By this, I mean advice or help with breastfeeding. I know some of these are difficult to remember, but please try to tell me what you do remember as it will be very useful in checking the quality of care provided in this facility.  *Sasa, nitakuuliza maswali zaidi kuhusu vipengele maalum vya ushauri nasaha waunyonyeshaji na usaidizi uliopokea kwa wahudumu wa kituo cha afya cha Mbagathi baada ya kujifungua [BABY’S NAME]. Kwa hili na maanisha ushauri au msaada wa kunyonyesha. najua baadhi ya haya ni magumu kukumbuka lakini tafadhali kuniambia unachokumbuka kwa sababu itakuwa muhimu sana katika kuanaglaia ubora wa huduma zinazotolewa katika kituo hiki.* | | |
| 10. In total, how many antenatal care visits have you attended while pregnant with your new baby? [DO NOT READ RESPONSE OPTIONS.]  *Kwa ujumla, ni kliniki ngapi za uja uzito umehudhuria wakati wa huu uja uzito?* [DO NOT READ RESPONSE OPTIONS.] | NUMBER: __________  DK/CAN'T REMEMBER 8 →  PREFER NOT TO ANSWER 9 → | IF ZERO SKIP TO Q12. |
| 11. How many of those visits were at Mbagathi? [DO NOT READ RESPONSE OPTIONS.]  *Ni kliniki kama ngapi hivi ulizohudhuria kwenye hospitali ya rufaa ya Mbagathi?* [DO NOT READ RESPONSE OPTIONS.] | NUMBER: __________  DK/CAN'T REMEMBER 8 →  PREFER NOT TO ANSWER 9 → |  |
| 1. After arriving in the postnatal care unit, about how long was it until any health facility staff gave you breastfeeding counseling and support or talked to you about or helped you with breastfeeding [BABY'S NAME]? [DO NOT READ RESPONSE OPTIONS.] *Baada ya kufika katika kitengo cha utunzaji baada ya kuzaa, ni muda gani ulichukua mfanyakazi yeyote wa afya akupe ushauri na usaidizi wa kunyonyesha au kuzungumza nawe kuhusu au kukusaidia katika kunyonyesha.* [DO NOT READ RESPONSE OPTIONS.] | < 1 HOUR 1  1–2 HOURS 2  3–4 HOURS 3  ≥ 5 HOURS 4  DK/CAN'T REMEMBER 8  PREFER NOT TO ANSWER 9 |  |
| 1. After giving birth to [BABY'S NAME] at Mbagathi, did you want to have someone with you such as a family member or friend when you received breastfeeding counseling and support? [DO NOT READ RESPONSE OPTIONS.] *Baada ya kujifungua [BABY'S NAME] huko mbagathi, Ulitaka kuwa na mtu pamoja nawe kama mtu wa familia au rafiki, ulipopata ushauri na usaidizi wa kunyonyesha?* [DO NOT READ RESPONSE OPTIONS.] | YES 1  NO 2  DK/CAN'T REMEMBER 8  PREFER NOT TO  ANSWER 9 |  |
| 1. Was someone with you when you received breastfeeding counseling? [DO NOT READ RESPONSE OPTIONS.] *Kulikuwa na mtu pamoja nawe ulipopokea ushauri wa kunyonyesha?* [DO NOT READ RESPONSE OPTIONS.] | YES 1 →  NO 2 →  DK/CAN'T REMEMBER 8 →  PREFER NOT TO ANSWER 9 → | GO TO Q16  GO TO Q15  GO TO Q17  GO TO Q17 |
| 1. What is the main reason why someone was not with you when you received breastfeeding counseling? [DO NOT READ RESPONSE OPTIONS. CHECK THE MOST APPROPRIATE RESPONSE OPTION.] *Ni nini sababu kuu ya mtu kutokuwa nawe wakati ulipopokea ushauri wa kunyonyesha?* [DO NOT READ RESPONSE OPTIONS. CHECK THE MOST APPROPRIATE RESPONSE OPTION.] | IT WAS NOT VISITING HOURS 1  WAS NOT ALLOWED 2  PERSON WAS NOT AVAILABLE 3  OTHER REASON 4  DK/CAN'T REMEMBER 8  PREFER NOT TO ANSWER 9 | GO TO Q17 |
| 1. Who was with you when you received breastfeeding counseling? [DO NOT READ RESPONSE OPTIONS. CHECK ALL THAT APPLY.] *Nani alikuwa nawe ulipopata ushauri wa kunyonyesha?*  [DO NOT READ RESPONSE OPTIONS. CHECK ALL THAT APPLY.] | HUSBAND OR PARTNER 1  MOTHER 2  MOTHER-IN-LAW 3  CHILD 4  OTHER FAMILY MEMBER 5  FRIEND 6  SOMEONE ELSE 7  DK/CAN'T REMEMBER 8  PREFER NOT TO ANSWER 9 |  |
| 1. Did the health facility staff who gave you breastfeeding counseling and support discuss how the person who was with you or another person could support you with breastfeeding? [DO NOT READ RESPONSE OPTIONS.]   *Je, yule mhudumu wa afya aliyekupea ushauri kuhusu kunyonyesha alizungumzia jinsi yule mtu ulikuwa naye angekusaidia kwa kunyonyesha?* [DO NOT READ RESPONSE OPTIONS.] | YES 1  NO 2  DK/CAN'T REMEMBER 8  PREFER NOT TO ANSWER 9 |  |
| 1. Did the health facility staff who gave you breastfeeding counseling and support after giving birth call you by your name or your child’s name? IF YES, ASK: Would you say this was all of the time, most of the time, or a few times? *Wahudumu wa kituo cha afya waliotoa ushauri na usaidizi wa kunyonyesha baada ya kujifungua, na walikuita kwa jina lako ama la mtoto wako?* *IWAPO NDIO, ULIZIA: Je, unaweza kusema hii ilikuwa mara chache, mara nyingi, au wakati wote ulipokuwa hospitalini?* | YES, ALL OF THE TIME 4  YES, MOST OF THE TIME 3  YES, A FEW TIMES 2  NO, NEVER 1  DK/CAN'T REMEMBER 8  PREFER NOT TO ANSWER 9 |  |
| 1. Did the health facility staff who gave you breastfeeding counseling and support treat you with respect or in a respectful manner? IF YES, ASK: Would you say this was all of the time, most of the time, or a few times? *Ukifiria wakati ulipokuwa kwenye hospitali ya mbagathi,* *wahudumu wa kituo cha afya waliokupa ushauri na usaidizi wa kunyonyesha walikuheshimu?* *IWAPO NDIO, ULIZIA: Je, unaweza kusema hii ilikuwa mara chache, mara nyingi, au wakati wote ulipokuwa hospitalini?* | YES, ALL OF THE TIME 4  YES, MOST OF THE TIME 3  YES, A FEW TIMES 2  NO, NEVER 1  DK/CAN'T REMEMBER 8  PREFER NOT TO ANSWER 9 |  |
| 1. Did the health facility staff who gave you breastfeeding counseling and support treat you in a friendly manner?  IF YES, ASK: Would you say this was all of the time, most of the time, or a few times? *Je, ulihisi wahudumu waliokupea ushauri na msaada wa kunyonyesha walikubeba kwa njia ya kirafiki?*   *IWAPO NDIO, ULIZIA: Je, unaweza kusema hii ilikuwa mara chache, mara nyingi, au wakati wote ulipokuwa hospitalini* | YES, ALL OF THE TIME 4  YES, MOST OF THE TIME 3  YES, A FEW TIMES 2  NO, NEVER 1  DK/CAN'T REMEMBER 8  PREFER NOT TO ANSWER 9 |  |
| 1. After giving birth to [BABY'S NAME] at Mbagathi, did you feel you could talk privately with the health facility staff who gave you breastfeeding counseling and support? In other words, could you speak without others overhearing your conversations? IF YES, ASK: Would you say this was all of the time, most of the time, or a few times? *Ulihisi unaweza kuzungumza na wahudumu wa kituo cha afya waliokupa ushauri wa kunyonyesha baada ya kujifungua [BABY'S NAME] bila wengine kutohusika katika uangalizi wako na kusikiliza mazungumzo yako? IWAPO NDIO, TAFUTA MAELEZO ZAIDI: Unaweza kusema hii ilikuwa ni kila mara, mara kadhaa ama mara chache wakati wako hospitalini?* | YES, ALL OF THE TIME 4  YES, MOST OF THE TIME 3  YES, A FEW TIMES 2  NO, NEVER 1  DK/CAN'T REMEMBER 8  PREFER NOT TO ANSWER 9 |  |
| 1. Did you feel you could ask the health facility staff who gave you breastfeeding counseling and support any questions you had about feeding your infant? IF YES, ASK: Would you say this was all of the time, most of the time, or a few times? *Ulihisi unaweza kumuuliza mhudumu wa kituo cha afya aliyekupea msaada na ushauri wa kunyonyesha, swali lolote kuhusu kulisha mtoto wako mchanga? IWAPO NDIO, ULIZIA: Je, unaweza kusema hii ilikuwa mara chache, mara nyingi, au wakati wote ulipokuwa hospitalini?* | YES, ALL OF THE TIME 4  YES, MOST OF THE TIME 3  YES, A FEW TIMES 2  NO, NEVER 1  DK/CAN'T REMEMBER 8  PREFER NOT TO ANSWER 9 |  |
| 1. Did the health facility staff who gave you breastfeeding counseling and support ask you how you were feeling? IF YES, ASK: Would you say this was all of the time, most of the time, or a few times? *Mhudumu wa kituo cha afya aliyekupea msaada na ushauri kuhusu kunyonyesha aliongea na wewe jinsi ulivyokuwa ukijisikia? IWAPO NDIO, ULIZIA: Je, unaweza kusema hii ilikuwa mara chache, mara nyingi, au wakati wote ulipokuwa hospitalini?* | YES, ALL OF THE TIME 4  YES, MOST OF THE TIME 3  YES, A FEW TIMES 2  NO, NEVER 1  DK/CAN'T REMEMBER 8  PREFER NOT TO ANSWER 9 |  |
| 1. After giving birth to [BABY'S NAME] at Mbagathi, did you feel the health facility staff who gave you breastfeeding counseling and support paid attention to you and your questions and concerns? IF YES, ASK: Would you say this was all of the time, most of the time, or a few times? *Wakati ulihitaji usaidizi, wa kunyonyesha ulihisi kwamba yule mhudumu wa kituo cha afya aliyekupea ushauri na msaada wa kunyonyesha alikusikiliza kwa makiniIWAPO NDIO, ULIZIA: Je, unaweza kusema hii ilikuwa mara chache, mara nyingi, au wakati wote ulipokuwa hospitalini?* | YES, ALL OF THE TIME 4  YES, MOST OF THE TIME 3  YES, A FEW TIMES 2  NO, NEVER 1  DK/CAN'T REMEMBER 8  PREFER NOT TO ANSWER 9 |  |
| 1. Did you feel the health facility staff who gave you breastfeeding counseling and support took the best care of you that they could? IF YES, ASK: Would you say this was all of the time, most of the time, or a few times?   *Ulihisi muhudumu wa kituo cha afya aliyekupea msaada na ushauri wa kunyonyesha alikutunza bora zaidi alivyoweza?* *IWAPO NDIO, ULIZIA: Je, unaweza kusema hii ilikuwa mara chache, mara nyingi, au wakati wote ulipokuwa hospitalini?* | YES, ALL OF THE TIME 4  YES, MOST OF THE TIME 3  YES, A FEW TIMES 2  NO, NEVER 1  DK/CAN'T REMEMBER 8  PREFER NOT TO ANSWER 9 |  |
| 1. Was a cloth, blanket, or screen available to use so that you did not feel physically exposed when breastfeeding? IF YES, ASK: Would you say this was all of the time, most of the time, or a few times? *Kulikuwa na kitamba, blanketi au kizuizi cha kutumia ili usijisikie wazi wakati wa kunyonyesha? IWAPO NDIO, ULIZIA: Je, unaweza kusema hii ilikuwa mara chache, mara nyingi, au wakati wote ulipokuwa hospitalini?* | YES, ALL OF THE TIME 4  YES, MOST OF THE TIME 3  YES, A FEW TIMES 2  NO, NEVER 1  DK/CAN'T REMEMBER 8  PREFER NOT TO ANSWER 9 |  |
| 1. Did the health facility staff who gave you breastfeeding counseling and support ask your permission or consent before helping or observing you breastfeed? IF YES, ASK: Would you say this was all of the time, most of the time, or a few times? *Yule muhudumu wa kituo cha afya aliyekupatia msaada na ushauri wa kunyonyesha alikuomba ruhusa kabla akusaidie ama kutazama vile unavyonyonyesha?* *IWAPO NDIO, ULIZIA: Je, unaweza kusema hii ilikuwa mara chache, mara nyingi, au wakati wote ulipokuwa hospitalini?* | YES, ALL OF THE TIME 4  YES, MOST OF THE TIME 3  YES, A FEW TIMES 2  NO, NEVER 1  DK/CAN'T REMEMBER 8  PREFER NOT TO ANSWER 9 |  |
| 1. Were you in any way unhappy with how health facility staff who gave you breastfeeding counseling and support treated you? [DO NOT READ RESPONSE OPTIONS.] *Kwa namna yeyote unaweza kuwa hukufurahishwa na jinsi wahudumu wa kituo cha afya waliokupa ushauri na usaidizi wa kunyonyesha, walivyo kutendea?* [DO NOT READ RESPONSE OPTIONS.] | YES 1  NO 2 →  DK 8 →  PREFER NOT TO ANSWER 9 → | GO TO Q27  GO TO Q28  GO TO Q28  GO TO Q28 |
| 1. Why were you unhappy with how health facility staff who gave you breastfeeding counseling and support treated you? How did they treat you? Kwa nini hukufurahishwa na jinsi wahudumu wa kituo cha afya waliokupa ushauri jinsi ya kunyonyesha na usaidizi walivyo kuchukulia |  |  |
| 1. Would you say that the health facility staff who gave you breastfeeding counseling and support treated you differently because of any personal attribute, like your age, marital status, number of children, education, wealth, or something like that? IF YES, ASK: Would you say this was all of the time, most of the time, or a few times? *Unaweza kusema kwamba wafanyi kazi wa kituo cha afya waliokupa ushauri na usaidizi wa kunyonyesha walikuchukulia tofauti kwa sababu ya sifa zozote ya kibinafsi kama vile umri wako, hali ya ndoa, idadi ya watoto, elimu, utajiri na mambo kama hayo?*   *IWAPO NDIO, ULIZIA: Je, unaweza kusema hii ilikuwa mara chache, mara nyingi, au wakati wote ulipokuwa hospitalini?* | YES, ALL OF THE TIME 4  YES, MOST OF THE TIME 3  YES, A FEW TIMES 2  NO, NEVER 1  DK/CAN'T REMEMBER 8  PREFER NOT TO ANSWER 9 |  |
| 1. Did you feel like you were physically mistreated, for instance, were you pushed, slapped, pinched, or physically mistreated in any other way specifically by the health facility staff who gave you breastfeeding counseling and support? IF YES, ASK: Would you say this was all of the time, most of the time, or a few times? *Je, ulihisi ulichukuliwa visivyo, kwa mfano ukasukumwa, ukapigwa kofi, kufinywa, ama dhuluma zinginezo za kimwili haswa na mhudumu wa kituo cha afya aliyekupea ushauri na msaada wa kunyonyesha? IWAPO NDIO, ULIZIA: Je, unaweza kusema hii ilikuwa mara chache, mara nyingi, au wakati wote ulipokuwa hospitalini?* | YES, ALL OF THE TIME 4  YES, MOST OF THE TIME 3  YES, A FEW TIMES 2  NO, NEVER 1  DK/CAN'T REMEMBER 8  PREFER NOT TO  ANSWER 9 |  |
| 1. Did you feel like you were verbally mistreated by the health facility staff who gave you breastfeeding counseling and support? For instance, were you shouted at, insulted, threatened, talked to rudely, or verbally mistreated in any other way? IF YES, ASK: Would you say this was all of the time, most of the time, or a few times? *Je, ulihisi yule mhudumu wa kituo cha afya aliyekupatia ushauri na usaidizi wa kunyonyesha alikufokea,karipia, kutusi,kutisha, alikuongelesha kwa madharau, ama alikudunisha kwa njia yoyote kwa matamshi? IWAPO NDIO, ULIZIA: Je, unaweza kusema hii ilikuwa mara chache, mara nyingi, au wakati wote ulipokuwa hospitalini?* | YES, ALL OF THE TIME 4  YES, MOST OF THE  TIME 3  YES, A FEW TIMES 2  NO, NEVER 1  DK/CAN'T REMEMBER 8  PREFER NOT TO ANSWER 9 |  |
| 1. Do you think the breastfeeding counseling and support that you were provided after delivering [BABY'S NAME] was helpful? [DO NOT READ RESPONSE OPTIONS.]   *Je, unafikiria ushauri wa kunyonyesha uliopewa baada ya kujifungua [BABY'S NAME] ulikuwa wa manufaa?* [DO NOT READ RESPONSE OPTIONS.] | YES 1 NO 2  DK/CAN'T REMEMBER 8  PREFER NOT TO ANSWER 9 | GO TO Q31a GO TO Q31b  GO TO Q32  GO TO Q32 |
| 31(a) How was it helpful? [DO NOT READ RESPONSE OPTIONS. AS THE MOTHER RESPONDS, CHECK OFF THE RESPONSE(S) THAT MOST CLOSELY RESEMBLES WHAT SHE HAS SAID. FOR ALL OTHER RESPONSES NOT LISTED, RECORD HER RESPONSE UNDER “OTHER.” IF YOU ARE UNSURE, RECORD RESPONSES UNDER “OTHER.”] PROBE: Was it helpful in any other way? *Ilikusaidia vipi?* [DO NOT READ RESPONSE OPTIONS. AS THE MOTHER RESPONDS, CHECK OFF THE RESPONSE(S) THAT MOST CLOSELY RESEMBLES WHAT SHE HAS SAID. FOR ALL OTHER RESPONSES NOT LISTED, RECORD HER RESPONSE UNDER “OTHER.” IF YOU ARE UNSURE, RECORD RESPONSES UNDER “OTHER.”]  *TAFUTA MAELEZO ZAIDI: Ilikusaidia kwa njia nyengineyo?* | THE HEALTH WORKER TAUGHT ME INFORMATION A  THE HEALTH WORKER HELPED ME GET MY BABY TO LATCH B  THE HEALTH WORKER HELPED ME POSITION MY BABY FOR BREASTFEEDING C  OTHER H  SPECIFY: _________ |  |
| 31(b) Why do you feel the breastfeeding counseling wasn’t helpful? [DO NOT READ RESPONSE OPTIONS. AS THE MOTHER RESPONDS, CHECK OFF THE RESPONSE(S) THAT MOST CLOSELY RESEMBLES WHAT SHE HAS SAID. FOR ALL OTHER RESPONSES NOT LISTED, RECORD HER RESPONSE UNDER “OTHER.” IF YOU ARE UNSURE, RECORD RESPONSES UNDER “OTHER.”] PROBE: Is there any other reason that you do not feel that it was helpful? *Kwa nini unahisi ushauri wa kunyonyesha haukuwa wa manufaa?* [DO NOT READ RESPONSE OPTIONS. AS THE MOTHER RESPONDS, CHECK OFF THE RESPONSE(S) THAT MOST CLOSELY RESEMBLES WHAT SHE HAS SAID. FOR ALL OTHER RESPONSES NOT LISTED, RECORD HER RESPONSE UNDER “OTHER.” IF YOU ARE UNSURE, RECORD RESPONSES UNDER “OTHER.”] *TAFUTA MAELEZO ZAIDI: Kuna sababu nyengine inayokufanya uhisi haukuwa na manufaa?* | I ALREADY KNEW EVERYTHING I NEEDED TO KNOW L  THE HEALTH WORKER DID NOT KNOW WHAT THEY WERE DOING M  THE HEALTH WORKER DID NOT EXPLAIN THINGS WELL N  I WAS NOT ABLE TO LATCH AND/OR POSITION MY BABY WELL O  OTHER S  SPECIFY: _________ |  |
| 1. Overall, were you satisfied or dissatisfied with the breastfeeding counseling that you were provided after delivering [BABY'S NAME]? IF SATISFIED, PROBE: Somewhat or very satisfied? IF DISSATISFIED, PROBE: Were you very dissatisfied, somewhat dissatisfied? *Kwa ujumla, uliridhika vipi na ule ushauri wakunyonyesha uliopewa baada ya kujifungua [BABY'S NAME]?* KAMA ULIRIDHIKA, NI KWA KIASI KIPI: uliridhika kabisa ama uliridhika kwa kadri? KAMA HUJARIDHIKA, NI KWA KIASI KIPI: *Sijaridhika kwa kadri na sijaridhika kabisa?* | VERY SATISFIED 1  SOMEWHAT SATISFIED 2  NEUTRAL 3  SOMEWHAT DISSATISFIED 4  VERY DISSATISFIED 5  DK/CAN'T REMEMBER 8  PREFER NOT TO ANSWER 9 |  |
| 1. Before we end, are you still breastfeeding [BABY'S NAME]? [DO NOT READ RESPONSE OPTIONS.]   *Kabla tumalize, bado unamnyonyesha [BABY'S NAME]?* [DO NOT READ RESPONSE OPTIONS.] | YES 1  NO 2  PREFER NOT TO ANSWER 9 |  |
| 1. Is there anything else you would like to tell me today about the breastfeeding counseling and support you received after delivering [BABY'S NAME]?   *Na kuna jambo lingine ungependa kuniambia kuhusu ushauri na usaidizi wa kunyonyesha?* RECORD RESPONSE. IF NOTHING ELSE, RECORD “NOTHING ELSE.” |  |  |

#### S6 Client Exit Interview Guide: Postpartum Women

***Mwongozo wa Mahojiano kwa Akina Mama Baada ya Kujifungua Wakitoka Hospitalini***

**Prior to the interview, record the following information:**

***Kabla ya mahojiano, rekodi habari zifuatazo:***

| CODE | AUTO-GENERATED |
| --- | --- |
| DATE | AUTO-GENERATED |
| INTERVIEWER | DROP-DOWN |

RESEARCHER CONFIRMS THAT IT IS THE CORRECT WOMAN, CONFIRMS LANGUAGE PREFERENCE, AND SEEKS INFORMED CONSENT.

IN WHICH LANGUAGE DOES THE RESPONDENT PREFER TO SPEAK? English Kiswahili

DID THE RESPONDENT GIVER HER CONSENT TO PARTICIPATE IN THIS INTERVIEW? Yes No

**IF RESPONDENT DOES NOT GIVE CONSENT, THANK HER FOR HER TIME AND END THE INTERVIEW.**

**IF RESPONDENT CONSENTS TO PARTICIPATE, PROCEED TO THE INTERVIEW.**

| **QUESTION** | **CODING CLASSIFICATION** | **SKIP** |
| --- | --- | --- |
| As a reminder, if, at any time, you do not want to answer a question or discuss an issue, you are free to decline to do so. You are also free to stop the interview at any time. The decision about whether or not to answer any specific question will not affect the services you receive at any health facility today or any time in the future.  *Ikiwa, wakati wowote, hutaki kujibu swali au kujadili suala, uko huru kukataa kufanya hivyo. Uko huru pia kujiondoa kwenye utafiti wakati wowote. Uamuzi kuhusu kushiriki au kutoshiriki katika utafiti huu au kujibu swali lolote maalumu hautaathiri haki zako za kupokea huduma zozote katika vituo vyovyote vya afya leo au wakati wowote ujao.* | | |
| 1. First, what is your new baby’s name? I will not write this down. I just want to be able to refer to her or him by name.   *Jina la mtoto aliyejifungua ni lipi? sitaandika jina lake nataka tu kurejelea kwa jina.* | DO NOT RECORD THE NAME.  NO NAME YET 1  PREFER NOT TO ANSWER 9 | . |
| 1. Next, how old were you at your last birthday? *Ulikuwa na umri gani katika siku yako ya kuzaliwa ya mwisho.* | ___ ___ years  DK 888 |  |
| 1. Have you ever been married? Are you currently married, living with a partner as if married, widowed, divorced, or separated? [DO NOT READ RESPONSE OPTIONS.]   *Je, kwa sasa umeolewa, unaishi na mwenzi wako kana kwamba umeolewa, umefiwa na mumewe/Mjane, umetalikiana, au umetengana?* [DO NOT READ RESPONSE OPTIONS.] | CURRENTLY MARRIED 2  LIVING WITH A PARTNER AS IF MARRIED 3  WIDOWED 4  DIVORCED OR SEPARATED 5  PREFER NOT TO ANSWER 9 |  |
| 1. Have you ever attended school? [DO NOT READ RESPONSE OPTIONS.]   *Uliwaahi kwenda shule kusoma?* [DO NOT READ RESPONSE OPTIONS.] | YES 1  NO 2 →  PREFER NOT TO ANSWER 9 → | GO TO Q6  GO TO Q6 |
| 1. What is the highest level of school you attended: primary, post-primary/vocational, secondary, college (middle level), university or higher?   *Ni kiwango gani cha juu zaidi cha shule ulichosoma: Shule ya msingi, baada ya shule ya msingi au ya ufundi, sekondari, chuo (kiwango cha kati), chuo kikuu au zaidi?* | PRIMARY 1  POST-PRIMARY/ VOCATIONAL 2  SECONDARY 3  COLLEGE (MIDDLE LEVEL) 4  UNIVERSITY OR HIGHER 5  PREFER NOT TO ANSWER 9 |  |
| 1. Aside from your own housework, have you done any work in the last 12 months? This includes a job for which you were paid in cash or kind. It includes small businesses or work on the family farm or in the family business. [DO NOT READ RESPONSE OPTIONS.] *Kando ya kazi zako za nyumba, uliwahi kufanya kazi ingine kwa kipindi cha miezi 12 iliyopita?* [DO NOT READ RESPONSE OPTIONS.] | YES 1  NO 2 →  PREFER NOT TO ANSWER 9 → | GO TO Q9  GO TO Q9 |
| 1. Are you paid in cash or kind? [DO NOT READ RESPONSE OPTIONS.] *Je, unalipwa pesa au kwa njia nyingine?*  [DO NOT READ RESPONSE OPTIONS.] | CASH ONLY 1  CASH AND KIND 2  IN KIND ONLY 3  NOT PAID 4  PREFER NOT TO ANSWER 9 |  |
| 1. What is your occupation? By this I mean, what kind of work do you mainly do? [RECORD HER RESPONSE IN THE SPACE PROVIDED.] *Kazi yako ni nini? Kwa hili namaanisha, ni aina gani ya kazi unayofanya hasa?*  [RECORD HER RESPONSE IN THE SPACE PROVIDED.] |  |  |
| 1. Finally, how many children have you given birth to?   *Mwishowe, umejifungua watoto wangapi?* NOTE: THIS INCLUDES LIVING OR DEAD, LIVING WITH THEM OR AWAY FROM HOME. | # OF CHILDREN: ___ ___  DK 88  PREFER NOT TO ANSWER 99 |  |
| Now, I am going to ask you some additional questions about specific aspects of the breastfeeding counseling and support you received from health facility staff at Mbagathi after giving birth to [BABY'S NAME]. By this, I mean advice or help with breastfeeding. I know some of these are difficult to remember, but please try to tell me what you do remember as it will be very useful in checking the quality of care provided in this facility.  *Sasa, nitakuuliza maswali zaidi kuhusu vipengele maalum vya ushauri nasaha waunyonyeshaji na usaidizi uliopokea kwa wahudumu wa kituo cha afya cha Mbagathi baada ya kujifungua [BABY’S NAME]. Kwa hili na maanisha ushauri au msaada wa kunyonyesha. najua baadhi ya haya ni magumu kukumbuka lakini tafadhali kuniambia unachokumbuka kwa sababu itakuwa muhimu sana katika kuanaglaia ubora wa huduma zinazotolewa katika kituo hiki.* | | |
| 10. In total, how many antenatal care visits have you attended while pregnant with your new baby? [DO NOT READ RESPONSE OPTIONS.]  *Kwa ujumla, ni kliniki ngapi za uja uzito umehudhuria wakati wa huu uja uzito?* [DO NOT READ RESPONSE OPTIONS.] | NUMBER: __________  DK/CAN'T REMEMBER 8 →  PREFER NOT TO ANSWER 9 → | IF ZERO SKIP TO Q12. |
| 11. How many of those visits were at Mbagathi? [DO NOT READ RESPONSE OPTIONS.]  *Ni kliniki kama ngapi hivi ulizohudhuria kwenye hospitali ya rufaa ya Mbagathi?* [DO NOT READ RESPONSE OPTIONS.] | NUMBER: __________  DK/CAN'T REMEMBER 8 →  PREFER NOT TO ANSWER 9 → |  |
| 1. After arriving in the postnatal care unit, about how long was it until any health facility staff gave you breastfeeding counseling and support or talked to you about or helped you with breastfeeding [BABY'S NAME]? [DO NOT READ RESPONSE OPTIONS.] *Baada ya kufika katika kitengo cha utunzaji baada ya kuzaa, ni muda gani ulichukua mfanyakazi yeyote wa afya akupe ushauri na usaidizi wa kunyonyesha au kuzungumza nawe kuhusu au kukusaidia katika kunyonyesha.* [DO NOT READ RESPONSE OPTIONS.] | < 1 HOUR 1  1–2 HOURS 2  3–4 HOURS 3  ≥ 5 HOURS 4  DK/CAN'T REMEMBER 8  PREFER NOT TO ANSWER 9 |  |
| 1. After giving birth to [BABY'S NAME] at Mbagathi, did you want to have someone with you such as a family member or friend when you received breastfeeding counseling and support? [DO NOT READ RESPONSE OPTIONS.] *Baada ya kujifungua [BABY'S NAME] huko mbagathi, Ulitaka kuwa na mtu pamoja nawe kama mtu wa familia au rafiki, ulipopata ushauri na usaidizi wa kunyonyesha?* [DO NOT READ RESPONSE OPTIONS.] | YES 1  NO 2  DK/CAN'T REMEMBER 8  PREFER NOT TO ANSWER 9 |  |
| 1. Was someone with you when you received breastfeeding counseling? [DO NOT READ RESPONSE OPTIONS.] *Kulikuwa na mtu pamoja nawe ulipopokea ushauri wa kunyonyesha?* [DO NOT READ RESPONSE OPTIONS.] | YES 1 →  NO 2 →  DK/CAN'T REMEMBER 8 →  PREFER NOT TO ANSWER 9 → | GO TO Q16  GO TO Q15  GO TO Q17  GO TO Q17 |
| 1. What is the main reason why someone was not with you when you received breastfeeding counseling? [DO NOT READ RESPONSE OPTIONS. CHECK THE MOST APPROPRIATE RESPONSE OPTION.] *Ni nini sababu kuu ya mtu kutokuwa nawe wakati ulipopokea ushauri wa kunyonyesha?* [DO NOT READ RESPONSE OPTIONS. CHECK THE MOST APPROPRIATE RESPONSE OPTION.] | IT WAS NOT VISITING HOURS 1  WAS NOT ALLOWED 2  PERSON WAS NOT AVAILABLE 3  OTHER REASON 4  DK/CAN'T REMEMBER 8  PREFER NOT TO ANSWER 9 | GO TO Q17 |
| 1. Who was with you when you received breastfeeding counseling? [DO NOT READ RESPONSE OPTIONS. CHECK ALL THAT APPLY.] *Nani alikuwa nawe ulipopata ushauri wa kunyonyesha?*  [DO NOT READ RESPONSE OPTIONS. CHECK ALL THAT APPLY.] | HUSBAND OR PARTNER 1  MOTHER 2  MOTHER-IN-LAW 3  CHILD 4  OTHER FAMILY MEMBER 5  FRIEND 6  SOMEONE ELSE 7  DK/CAN'T REMEMBER 8  PREFER NOT TO ANSWER 9 |  |
| 1. Did the health facility staff who gave you breastfeeding counseling and support discuss how the person who was with you or another person could support you with breastfeeding? [DO NOT READ RESPONSE OPTIONS.]   *Je, yule mhudumu wa afya aliyekupea ushauri kuhusu kunyonyesha alizungumzia jinsi yule mtu ulikuwa naye angekusaidia kwa kunyonyesha?* [DO NOT READ RESPONSE OPTIONS.] | YES 1  NO 2  DK/CAN'T REMEMBER 8  PREFER NOT TO ANSWER 9 |  |
| 1. Did the health facility staff who gave you breastfeeding counseling and support after giving birth call you by your name or your child’s name? IF YES, ASK: Would you say this was all of the time, most of the time, or a few times? *Wahudumu wa kituo cha afya waliotoa ushauri na usaidizi wa kunyonyesha baada ya kujifungua, na walikuita kwa jina lako ama la mtoto wako?* *IWAPO NDIO, ULIZIA: Je, unaweza kusema hii ilikuwa mara chache, mara nyingi, au wakati wote ulipokuwa hospitalini?* | YES, ALL OF THE TIME 4  YES, MOST OF THE TIME 3  YES, A FEW TIMES 2  NO, NEVER 1  DK/CAN'T REMEMBER 8  PREFER NOT TO ANSWER 9 |  |
| 1. Did the health facility staff who gave you breastfeeding counseling and support treat you with respect or in a respectful manner? IF YES, ASK: Would you say this was all of the time, most of the time, or a few times? *Ukifiria wakati ulipokuwa kwenye hospitali ya mbagathi,* *wahudumu wa kituo cha afya waliokupa ushauri na usaidizi wa kunyonyesha walikuheshimu?* *IWAPO NDIO, ULIZIA: Je, unaweza kusema hii ilikuwa mara chache, mara nyingi, au wakati wote ulipokuwa hospitalini?* | YES, ALL OF THE TIME 4  YES, MOST OF THE TIME 3  YES, A FEW TIMES 2  NO, NEVER 1  DK/CAN'T REMEMBER 8  PREFER NOT TO ANSWER 9 |  |
| 1. Did the health facility staff who gave you breastfeeding counseling and support treat you in a friendly manner? IF YES, ASK: Would you say this was all of the time, most of the time, or a few times? *Je, ulihisi wahudumu waliokupea ushauri na msaada wa kunyonyesha walikubeba kwa njia ya kirafiki?*   *IWAPO NDIO, ULIZIA: Je, unaweza kusema hii ilikuwa mara chache, mara nyingi, au wakati wote ulipokuwa hospitalini* | YES, ALL OF THE TIME 4  YES, MOST OF THE TIME 3  YES, A FEW TIMES 2  NO, NEVER 1  DK/CAN'T REMEMBER 8  PREFER NOT TO ANSWER 9 |  |
| 1. After giving birth to [BABY'S NAME] at Mbagathi, did you feel you could talk privately with the health facility staff who gave you breastfeeding counseling and support? In other words, could you speak without others overhearing your conversations? IF YES, ASK: Would you say this was all of the time, most of the time, or a few times? *Ulihisi unaweza kuzungumza na wahudumu wa kituo cha afya waliokupa ushauri wa kunyonyesha baada ya kujifungua [BABY'S NAME] bila wengine kutohusika katika uangalizi wako na kusikiliza mazungumzo yako? IWAPO NDIO, TAFUTA MAELEZO ZAIDI: Unaweza kusema hii ilikuwa ni kila mara, mara kadhaa ama mara chache wakati wako hospitalini?* | YES, ALL OF THE TIME 4  YES, MOST OF THE TIME 3  YES, A FEW TIMES 2  NO, NEVER 1  DK/CAN'T REMEMBER 8  PREFER NOT TO ANSWER 9 |  |
| 1. Did you feel you could ask the health facility staff who gave you breastfeeding counseling and support any questions you had about feeding your infant? IF YES, ASK: Would you say this was all of the time, most of the time, or a few times? *Ulihisi unaweza kumuuliza mhudumu wa kituo cha afya aliyekupea msaada na ushauri wa kunyonyesha, swali lolote kuhusu kulisha mtoto wako mchanga? IWAPO NDIO, ULIZIA: Je, unaweza kusema hii ilikuwa mara chache, mara nyingi, au wakati wote ulipokuwa hospitalini?* | YES, ALL OF THE TIME 4  YES, MOST OF THE TIME 3  YES, A FEW TIMES 2  NO, NEVER 1  DK/CAN'T REMEMBER 8  PREFER NOT TO ANSWER 9 |  |
| 1. Did the health facility staff who gave you breastfeeding counseling and support ask you how you were feeling? IF YES, ASK: Would you say this was all of the time, most of the time, or a few times? *Mhudumu wa kituo cha afya aliyekupea msaada na ushauri kuhusu kunyonyesha aliongea na wewe jinsi ulivyokuwa ukijisikia? IWAPO NDIO, ULIZIA: Je, unaweza kusema hii ilikuwa mara chache, mara nyingi, au wakati wote ulipokuwa hospitalini?* | YES, ALL OF THE TIME 4  YES, MOST OF THE TIME 3  YES, A FEW TIMES 2  NO, NEVER 1  DK/CAN'T REMEMBER 8  PREFER NOT TO ANSWER 9 |  |
| 1. After giving birth to [BABY'S NAME] at Mbagathi, did you feel the health facility staff who gave you breastfeeding counseling and support paid attention to you and your questions and concerns? IF YES, ASK: Would you say this was all of the time, most of the time, or a few times? *Wakati ulihitaji usaidizi, wa kunyonyesha ulihisi kwamba yule mhudumu wa kituo cha afya aliyekupea ushauri na msaada wa kunyonyesha alikusikiliza kwa makini* *IWAPO NDIO, ULIZIA: Je, unaweza kusema hii ilikuwa mara chache, mara nyingi, au wakati wote ulipokuwa hospitalini?* | YES, ALL OF THE TIME 4  YES, MOST OF THE TIME 3  YES, A FEW TIMES 2  NO, NEVER 1  DK/CAN'T REMEMBER 8  PREFER NOT TO ANSWER 9 |  |
| 1. Did you feel the health facility staff who gave you breastfeeding counseling and support took the best care of you that they could? IF YES, ASK: Would you say this was all of the time, most of the time, or a few times?   *Ulihisi muhudumu wa kituo cha afya aliyekupea msaada na ushauri wa kunyonyesha alikutunza bora zaidi alivyoweza?* *IWAPO NDIO, ULIZIA: Je, unaweza kusema hii ilikuwa mara chache, mara nyingi, au wakati wote ulipokuwa hospitalini?* | YES, ALL OF THE TIME 4  YES, MOST OF THE TIME 3  YES, A FEW TIMES 2  NO, NEVER 1  DK/CAN'T REMEMBER 8  PREFER NOT TO ANSWER 9 |  |
| 1. Was a cloth, blanket, or screen available to use so that you did not feel physically exposed when breastfeeding? IF YES, ASK: Would you say this was all of the time, most of the time, or a few times? *Kulikuwa na kitamba, blanketi au kizuizi cha kutumia ili usijisikie wazi wakati wa kunyonyesha? IWAPO NDIO, ULIZIA: Je, unaweza kusema hii ilikuwa mara chache, mara nyingi, au wakati wote ulipokuwa hospitalini?* | YES, ALL OF THE TIME 4  YES, MOST OF THE TIME 3  YES, A FEW TIMES 2  NO, NEVER 1  DK/CAN'T REMEMBER 8  PREFER NOT TO ANSWER 9 |  |
| 1. Did the health facility staff who gave you breastfeeding counseling and support ask your permission or consent before helping or observing you breastfeedIF YES, ASK: Would you say this was all of the time, most of the time, or a few times? *Yule muhudumu wa kituo cha afya aliyekupatia msaada na ushauri wa kunyonyesha alikuomba ruhusa kabla akusaidie ama kutazama vile unavyonyonyesha?* *IWAPO NDIO, ULIZIA: Je, unaweza kusema hii ilikuwa mara chache, mara nyingi, au wakati wote ulipokuwa hospitalini?* | YES, ALL OF THE TIME 4  YES, MOST OF THE TIME 3  YES, A FEW TIMES 2  NO, NEVER 1  DK/CAN'T REMEMBER 8  PREFER NOT TO ANSWER 9 |  |
| 1. Were you in any way unhappy with how health facility staff who gave you breastfeeding counseling and support treated you? [DO NOT READ RESPONSE OPTIONS.] *Kwa namna yeyote unaweza kuwa hukufurahishwa na jinsi wahudumu wa kituo cha afya waliokupa ushauri na usaidizi wa kunyonyesha, walivyo kutendea?* [DO NOT READ RESPONSE OPTIONS.] | YES 1  NO 2 →  DK 8 →  PREFER NOT TO ANSWER 9 → | GO TO Q27  GO TO Q28  GO TO Q28  GO TO Q28 |
| 1. Why were you unhappy with how health facility staff who gave you breastfeeding counseling and support treated you? How did they treat you? Kwa nini hukufurahishwa na jinsi wahudumu wa kituo cha afya waliokupa ushauri jinsi ya kunyonyesha na usaidizi walivyo kuchukulia |  |  |
| 1. Would you say that the health facility staff who gave you breastfeeding counseling and support treated you differently because of any personal attribute, like your age, marital status, number of children, education, wealth, or something like that? IF YES, ASK: Would you say this was all of the time, most of the time, or a few times? *Unaweza kusema kwamba wafanyi kazi wa kituo cha afya waliokupa ushauri na usaidizi wa kunyonyesha walikuchukulia tofauti kwa sababu ya sifa zozote ya kibinafsi kama vile umri wako, hali ya ndoa, idadi ya watoto, elimu, utajiri na mambo kama hayo?*   *IWAPO NDIO, ULIZIA: Je, unaweza kusema hii ilikuwa mara chache, mara nyingi, au wakati wote ulipokuwa hospitalini?* | YES, ALL OF THE TIME 4  YES, MOST OF THE TIME 3  YES, A FEW TIMES 2  NO, NEVER 1  DK/CAN'T REMEMBER 8  PREFER NOT TO ANSWER 9 |  |
| 1. Did you feel like you were physically mistreated, for instance, were you pushed, slapped, pinched, or physically mistreated in any other way specifically by the health facility staff who gave you breastfeeding counseling and support? IF YES, ASK: Would you say this was all of the time, most of the time, or a few times? *Je, ulihisi ulichukuliwa visivyo, kwa mfano ukasukumwa, ukapigwa kofi, kufinywa, ama dhuluma zinginezo za kimwili haswa na mhudumu wa kituo cha afya aliyekupea ushauri na msaada wa kunyonyesha? IWAPO NDIO, ULIZIA: Je, unaweza kusema hii ilikuwa mara chache, mara nyingi, au wakati wote ulipokuwa hospitalini?* | YES, ALL OF THE TIME 4  YES, MOST OF THE TIME 3  YES, A FEW TIMES 2  NO, NEVER 1  DK/CAN'T REMEMBER 8  PREFER NOT TO  ANSWER 9 |  |
| 1. Did you feel like you were verbally mistreated by the health facility staff who gave you breastfeeding counseling and support? For instance, were you shouted at, insulted, threatened, talked to rudely, or verbally mistreated in any other way? IF YES, ASK: Would you say this was all of the time, most of the time, or a few times? *Je, ulihisi yule mhudumu wa kituo cha afya aliyekupatia ushauri na usaidizi wa kunyonyesha alikufokea,karipia, kutusi,kutisha, alikuongelesha kwa madharau, ama alikudunisha kwa njia yoyote kwa matamshi? IWAPO NDIO, ULIZIA: Je, unaweza kusema hii ilikuwa mara chache, mara nyingi, au wakati wote ulipokuwa hospitalini?* | YES, ALL OF THE TIME 4  YES, MOST OF THE TIME 3  YES, A FEW TIMES 2  NO, NEVER 1  DK/CAN'T REMEMBER 8  PREFER NOT TO ANSWER 9 |  |
| 1. Do you think the breastfeeding counseling and support that you were provided after delivering [BABY'S NAME] was helpful? [DO NOT READ RESPONSE OPTIONS.]   *Je, unafikiria ushauri wa kunyonyesha uliopewa baada ya kujifungua [BABY'S NAME] ulikuwa wa manufaa?* [DO NOT READ RESPONSE OPTIONS.] | YES 1 NO 2  DK/CAN'T REMEMBER 8  PREFER NOT TO ANSWER 9 | GO TO Q31aGO TO Q31b  GO TO Q32  GO TO Q32 |
| 31(a) How was it helpful? [DO NOT READ RESPONSE OPTIONS. AS THE MOTHER RESPONDS, CHECK OFF THE RESPONSE(S) THAT MOST CLOSELY RESEMBLES WHAT SHE HAS SAID. FOR ALL OTHER RESPONSES NOT LISTED, RECORD HER RESPONSE UNDER “OTHER.” IF YOU ARE UNSURE, RECORD RESPONSES UNDER “OTHER.”] PROBE: Was it helpful in any other way? *Ilikusaidia vipi?* [DO NOT READ RESPONSE OPTIONS. AS THE MOTHER RESPONDS, CHECK OFF THE RESPONSE(S) THAT MOST CLOSELY RESEMBLES WHAT SHE HAS SAID. FOR ALL OTHER RESPONSES NOT LISTED, RECORD HER RESPONSE UNDER “OTHER.” IF YOU ARE UNSURE, RECORD RESPONSES UNDER “OTHER.”]  *TAFUTA MAELEZO ZAIDI: Ilikusaidia kwa njia nyengineyo?* | THE HEALTH WORKER TAUGHT ME INFORMATION A  THE HEALTH WORKER HELPED ME GET MY BABY TO LATCH B  THE HEALTH WORKER HELPED ME POSITION MY BABY FOR BREASTFEEDING C  OTHER H  SPECIFY: _________ |  |
| 31(b) Why do you feel the breastfeeding counseling wasn’t helpful? [DO NOT READ RESPONSE OPTIONS. AS THE MOTHER RESPONDS, CHECK OFF THE RESPONSE(S) THAT MOST CLOSELY RESEMBLES WHAT SHE HAS SAID. FOR ALL OTHER RESPONSES NOT LISTED, RECORD HER RESPONSE UNDER “OTHER.” IF YOU ARE UNSURE, RECORD RESPONSES UNDER “OTHER.”] PROBE: Is there any other reason that you do not feel that it was helpful? *Kwa nini unahisi ushauri wa kunyonyesha haukuwa wa manufaa?* [DO NOT READ RESPONSE OPTIONS. AS THE MOTHER RESPONDS, CHECK OFF THE RESPONSE(S) THAT MOST CLOSELY RESEMBLES WHAT SHE HAS SAID. FOR ALL OTHER RESPONSES NOT LISTED, RECORD HER RESPONSE UNDER “OTHER.” IF YOU ARE UNSURE, RECORD RESPONSES UNDER “OTHER.”] *TAFUTA MAELEZO ZAIDI: Kuna sababu nyengine inayokufanya uhisi haukuwa na manufaa?* | I ALREADY KNEW EVERYTHING I NEEDED TO KNOW L  THE HEALTH WORKER DID NOT KNOW WHAT THEY WERE DOING M  THE HEALTH WORKER DID NOT EXPLAIN THINGS WELL N  I WAS NOT ABLE TO LATCH AND/OR POSITION MY BABY WELL O  OTHER S  SPECIFY: _________ |  |
| 1. Overall, were you satisfied or dissatisfied with the breastfeeding counseling that you were provided after delivering [BABY'S NAME]? IF SATISFIED, PROBE: Somewhat or very satisfied? IF DISSATISFIED, PROBE: Were you very dissatisfied, somewhat dissatisfied? *Kwa ujumla, uliridhika vipi na ule ushauri wakunyonyesha uliopewa baada ya kujifungua [BABY'S NAME]?* KAMA ULIRIDHIKA, NI KWA KIASI KIPI: uliridhika kabisa ama uliridhika kwa kadri? KAMA HUJARIDHIKA, NI KWA KIASI KIPI: *Sijaridhika kwa kadri na sijaridhika kabisa?* | VERY SATISFIED 1  SOMEWHAT  SATISFIED 2  NEUTRAL 3  SOMEWHAT DISSATISFIED 4  VERY DISSATISFIED 5  DK/CAN'T REMEMBER 8  PREFER NOT TO ANSWER 9 |  |
| 1. Before we end, are you still breastfeeding [BABY'S NAME]? [DO NOT READ RESPONSE OPTIONS.]   *Kabla tumalize, bado unamnyonyesha [BABY'S NAME]?* [DO NOT READ RESPONSE OPTIONS.] | YES 1  NO 2  PREFER NOT TO ANSWER 9 |  |
| 1. Is there anything else you would like to tell me today about the breastfeeding counseling and support you received after delivering [BABY'S NAME]?   *Na kuna jambo lingine ungependa kuniambia kuhusu ushauri na usaidizi wa kunyonyesha?* RECORD RESPONSE. IF NOTHING ELSE, RECORD “NOTHING ELSE.” |  |  |

Thank you so much for your time!

*Asante sana kwa wakati wako!*

#### S7 Client Exit Interview Guide–Antenatal Care Clinic

**PRIOR TO THE INTERVIEW, RECORD THE FOLLOWING INFORMATION:**

| CODE/*KODI* | AUTO-GENERATED |
| --- | --- |
| DATE/*TAREHE* | AUTO-GENERATED |
| INTERVIEWER | DROP-DOWN |

RESEARCHER CONFIRMS THAT IT IS THE CORRECT WOMAN, CONFIRMS LANGUAGE PREFERENCE, AND SEEKS INFORMED CONSENT.

IN WHICH LANGUAGE DOES THE RESPONDENT PREFER TO SPEAK?

English Kiswahili

DID THE RESPONDENT GIVE HER CONSENT TO PARTICIPATE IN THIS INTERVIEW? Yes No

**IF RESPONDENT DOES NOT GIVE CONSENT, THANK HER FOR HER TIME AND END THE INTERVIEW.**

**IF RESPONDENT CONSENTS TO PARTICIPATE, PROCEED TO THE INTERVIEW.**

| **QUESTION** | **CODING CLASSIFICATION** | **SKIP** |
| --- | --- | --- |
| As a reminder, if, at any time, you do not want to answer a question or discuss an issue, you are free to decline to do so. You are also free to stop the interview at any time. The decision about whether or not to answer any specific question will not affect the services you receive at any health facility today or any time in the future.  *Ikiwa, wakati wowote, hutaki kujibu swali au kujadili suala, uko huru kukataa kufanya hivyo. Uko huru pia kujiondoa kwenye utafiti wakati wowote. Uamuzi kuhusu kushiriki au kutoshiriki katika utafiti huu au kujibu swali lolote maalumu hautaathiri haki zako za kupokea huduma zozote katika vituo vyovyote vya afya leo au wakati wowote ujao.* | | |
| 1. First, can you confirm that you are still pregnant? [DO NOT READ RESPONSE OPTIONS.]   *Kwanza, unaweza kudhibitisha kwamba uko na uja uzito?* [DO NOT READ RESPONSE OPTIONS.] | YES 1  NO 2 →  PREFER NOT TO ANSWER 9 → | THANK THE WOMAN, EXPLAIN THAT OUR STUDY IS ABOUT PREGNANT WOMEN SO SHE IS NOT ELIGIBLE, AND END THE INTERVIEW. |
| 1. In total, how many antenatal care visits have you attended during this pregnancy? [DO NOT READ RESPONSE OPTIONS.]   *Kwa ujumla, ni kliniki ngapi za uja uzito umehudhuria wakati wa huu uja uzito?* [DO NOT READ RESPONSE OPTIONS.] | NUMBER: __________  DK/CAN'T REMEMBER 8 →  PREFER NOT TO ANSWER 9 → |  |
| 1. How many of those visits were at Mbagathi? [DO NOT READ RESPONSE OPTIONS.]   *Ni kliniki kama ngapi hivi ulizohudhuria kwenye hospitali ya rufaa ya Mbagathi?* [DO NOT READ RESPONSE OPTIONS.] | NUMBER: __________  DK/CAN'T REMEMBER 8 →  PREFER NOT TO ANSWER 9 → |  |
| 1. Next, how old were you at your last birthday? *Ulikuwa na umri gani katika siku yako ya kuzaliwa ya mwisho?* | ___ ___ years  DK 888 |  |
| 1. Have you ever been married? Are you currently married, living with a partner as if married, widowed, divorced, or separated? [DO NOT READ RESPONSE OPTIONS.]   *Je, kwa sasa umeolewa, unaishi na mwenzi wako kana kwamba umeolewa, umefiwa na mumewe/Mjane, umetalikiana, au umetengana?* [DO NOT READ RESPONSE OPTIONS.] | CURRENTLY MARRIED 2  LIVING WITH A PARTNER AS IF MARRIED 3  WIDOWED 4  DIVORCED OR SEPARATED 5  PREFER NOT TO ANSWER 9 |  |
| 1. Have you ever attended school? [DO NOT READ RESPONSE OPTIONS.]   *Uliwaahi kwenda shule kusoma?* [DO NOT READ RESPONSE OPTIONS.] | YES 1  NO 2 →  PREFER NOT TO ANSWER 9 → | GO TO Q8  GO TO Q8 |
| 1. What is the highest level of school you attended: primary, post-primary/vocational, secondary, college (middle level), university or higher?   *Ni kiwango gani cha juu zaidi cha shule ulichosoma: Shule ya msingi, baada ya shule ya msingi au ya ufundi, sekondari, chuo (kiwango cha kati), chuo kikuu au zaidi?* | PRIMARY 1  POST-PRIMARY/ VOCATIONAL 2  SECONDARY 3  COLLEGE (MIDDLE LEVEL) 4  UNIVERSITY OR HIGHER 5  PREFER NOT TO ANSWER 9 |  |
| 1. Aside from your own housework, have you done any work in the last 12 months? This includes a job for which you were paid in cash or kind. It includes small businesses or work on the family farm or in the family business. [DO NOT READ RESPONSE OPTIONS.] *Kando ya kazi zako za nyumba, uliwahi kufanya kazi ingine kwa kipindi cha miezi 12 iliyopita?* [DO NOT READ RESPONSE OPTIONS.] | YES 1  NO 2 →  PREFER NOT TO ANSWER 9 → | GO TO Q11  GO TO Q11 |
| 1. Are you paid in cash or kind? [DO NOT READ RESPONSE OPTIONS.] *Je, unalipwa pesa au kwa njia nyingine?*  [DO NOT READ RESPONSE OPTIONS.] | CASH ONLY 1  CASH AND KIND 2  IN KIND ONLY 3  NOT PAID 4  PREFER NOT TO ANSWER 9 |  |
| 1. What is your occupation? By this I mean, what kind of work do you mainly do? [RECORD HER RESPONSE IN THE SPACE PROVIDED.] *Kazi yako ni nini? Kwa hili namaanisha, ni aina gani ya kazi unayofanya hasa?*  [RECORD HER RESPONSE IN THE SPACE PROVIDED.] |  |  |
| 1. Finally, how many children have you given birth to? [THIS INCLUDES LIVING OR DEAD, LIVING WITH THEM OR AWAY FROM HOME.]   *Mwishowe, umejifungua watoto wangapi?* [THIS INCLUDES LIVING OR DEAD, LIVING WITH THEM OR AWAY FROM HOME.] | # OF CHILDREN: ___ ___  DK 88  PREFER NOT TO ANSWER 99 |  |
| Now I am going to ask you some more questions about specific aspects of the breastfeeding counseling you received during an antenatal visit from health facility staff in the last two weeks at Mbagathi. I know some of these are difficult to remember, but please try to tell me what you do remember as it will be very useful in checking the quality of care provided in this facility.  *Sasa, nitakuuliza maswali zaidi kuhusu vipengele maalum vya ushauri nasaha wa unyonyeshaji uliopokea kwa wahudumu wa kituo cha afya cha Mbagathi wakati wa ziara ya kliniki ya kabla ya kujifungua [BABY'S NAME]. Kwa hili na maanisha ushauri wa kunyonyesha. najua baadhi ya haya ni magumu kukumbuka lakini tafadhali kuniambia unachokumbuka kwa sababu itakuwa muhimu sana katika kuangalia ubora wa huduma zinazotolewa katika kituo hiki.* | | |
| 1. Did you want to have someone with you such as a family member or friend when you received breastfeeding counseling during your antenatal visit(s) at Mbagathi in the last two weeks? [DO NOT READ RESPONSE OPTIONS.] *Baada ya ziara yako ya kliniki ya kabla ya kujifungua huko mbagathi, Ulitaka kuwa na mtu pamoja nawe kama mtu wa familia au rafiki, ulipopata ushauri wa kunyonyesha?* [DO NOT READ RESPONSE OPTIONS.] | YES 1  NO 2  DK/CAN'T REMEMBER 8  PREFER NOT TO ANSWER 9 |  |
| 1. Was someone with you when you received breastfeeding counseling during your antenatal visit(s) at Mbagathi in the last two weeks? [DO NOT READ RESPONSE OPTIONS.] *Kulikuwa na mtu pamoja nawe ulipopokea ushauri wa kunyonyesha?* [DO NOT READ RESPONSE OPTIONS.] | YES 1 →  NO 2 →  DK/CAN'T REMEMBER 8 →  PREFER NOT TO ANSWER 9 → | GO TO Q15GO TO Q14  GO TO Q16  GO TO Q16 |
| 1. What is the main reason why someone was not with you when you received breastfeeding counseling during your antenatal visit(s) at Mbagathi in the last two weeks? [DO NOT READ RESPONSE OPTIONS. CHECK THE MOST APPROPRIATE RESPONSE OPTION.] *Ni nini sababu kuu ya mtu kutokuwa nawe wakati ulipopokea ushauri wa kunyonyesha?* [DO NOT READ RESPONSE OPTIONS. CHECK THE MOST APPROPRIATE RESPONSE OPTION.] | NOT ALLOWED, IT WAS NOT VISITING HOURS 1  NOT ALLOWED, SOME OTHER REASON 2  NOT AVAILABLE 3  DK/CAN'T REMEMBER 8  PREFER NOT TO ANSWER 9 |  |
| 1. Who was with you when you received breastfeeding counseling during your antenatal visit(s) at Mbagathi in the last two weeks? [DO NOT READ RESPONSE OPTIONS. CHECK ALL THAT APPLY.] *Nani alikuwa nawe ulipopata ushauri wa kunyonyesha?*  [DO NOT READ RESPONSE OPTIONS. CHECK ALL THAT APPLY.] | HUSBAND OR PARTNER 1  MOTHER 2  MOTHER-IN-LAW 3  CHILD 4  OTHER FAMILY MEMBER 5  FRIEND 6  SOMEONE ELSE 7  DK/CAN'T REMEMBER 8  PREFER NOT TO ANSWER 9 |  |
| 1. Did the health facility staff discuss how the person who was with you or another person could support you with breastfeeding when you received breastfeeding counseling during your antenatal visit(s) at Mbagathi in the last two weeks? [DO NOT READ RESPONSE OPTIONS.]   *Je, yule mhudumu wa afya aliyekupea ushauri kuhusu kunyonyesha alizungumzia jinsi yule mtu ulikuwa naye angekusaidia kwa kunyonyesha?* [DO NOT READ RESPONSE OPTIONS.] | YES 1  NO 2  DK/CAN'T REMEMBER 8  PREFER NOT TO ANSWER 9 |  |
| 1. Did the health facility staff call you by your name when you received breastfeeding counseling during your antenatal visit(s) at Mbagathi in the last two weeks? IF YES, ASK: Would you say this was all of the time, most of the time, or a few times? *Wahudumu wa kituo cha afya waliotoa ushauri na usaidizi wa kunyonyesha baada ya kujifungua, na walikuita kwa jina lako?* *IWAPO NDIO, ULIZIA: Je, unaweza kusema hii ilikuwa mara chache, mara nyingi, au wakati wote ulipokuwa hospitalini?* | YES, ALL OF THE TIME 4  YES, MOST OF THE TIME 3  YES, A FEW TIMES 2  NO, NEVER 1  DK/CAN'T REMEMBER 8  PREFER NOT TO ANSWER 9 |  |
| 1. Did the health facility staff treat you with respect or in a respectful manner when you received breastfeeding counseling during your antenatal visit(s) at Mbagathi in the last two weeks? IF YES, ASK: Would you say this was all of the time, most of the time, or a few times? *Ukifiria wakati ulipokuwa kwenye hospitali ya mbagathi,* *wahudumu wa kituo cha afya waliokupa ushauri wa kunyonyesha walikuheshimu?* *IWAPO NDIO, ULIZIA: Je, unaweza kusema hii ilikuwa mara chache, mara nyingi, au wakati wote ulipokuwa hospitalini?* | YES, ALL OF THE TIME 4  YES, MOST OF THE TIME 3  YES, A FEW TIMES 2  NO, NEVER 1  DK/CAN'T REMEMBER 8  PREFER NOT TO ANSWER 9 |  |
| Did the health facility staff treat you in a friendly manner when you received breastfeeding counseling during your antenatal visit(s) at Mbagathi in the last two weeks? IF YES, ASK: Would you say this was all of the time, most of the time, or a few times? *Je, ulihisi wahudumu waliokupea ushauri wa kunyonyesha walikubeba kwa njia ya kirafiki?IWAPO NDIO, ULIZIA: Je, unaweza kusema hii ilikuwa mara chache, mara nyingi, au wakati wote ulipokuwa hospitalini* | YES, ALL OF THE TIME 4  YES, MOST OF THE TIME 3  YES, A FEW TIMES 2  NO, NEVER 1  DK/CAN'T REMEMBER 8  PREFER NOT TO ANSWER 9 |  |
| 1. Did you feel you could ask the health facility staff any questions you had about feeding your infant when you received breastfeeding counseling during your antenatal visit(s) at Mbagathi in the last two weeks? IF YES, ASK: Would you say this was all of the time, most of the time, or a few times? *Ulihisi unaweza kumuuliza mhudumu wa kituo cha afya aliyekupea ushauri wa kunyonyesha, swali lolote kuhusu kulisha mtoto wako mchanga? IWAPO NDIO, ULIZIA: Je, unaweza kusema hii ilikuwa mara chache, mara nyingi, au wakati wote ulipokuwa hospitalini?* | YES, ALL OF THE TIME 4  YES, MOST OF THE  TIME 3  YES, A FEW TIMES 2  NO, NEVER 1  DK/CAN'T REMEMBER 8  PREFER NOT TO ANSWER 9 |  |
| 1. Did the health facility staff ask you about how you were feeling when you received breastfeeding counseling during your antenatal visit(s) at Mbagathi in the last two weeks? IF YES, ASK: Would you say this was all of the time, most of the time, or a few times? *Mhudumu wa kituo cha afya aliyekupea ushauri kuhusu kunyonyesha aliongea na wewe jinsi ulivyokuwa ukijisikia? IWAPO NDIO, ULIZIA: Je, unaweza kusema hii ilikuwa mara chache, mara nyingi, au wakati wote ulipokuwa hospitalini?* | YES, ALL OF THE TIME 4  YES, MOST OF THE TIME 3  YES, A FEW TIMES 2  NO, NEVER 1  DK/CAN'T REMEMBER 8  PREFER NOT TO ANSWER 9 |  |
| 1. Did you feel the health facility staff paid attention to you and your questions and concerns when you received breastfeeding counseling during your antenatal visit(s) at Mbagathi in the last two weeks? IF YES, ASK: Would you say this was all of the time, most of the time, or a few times? *Wakati ulihitaji usaidizi, wa kunyonyesha ulihisi kwamba yule mhudumu wa kituo cha afya aliyekupea ushauri wa kunyonyesha alikusikiliza kwa makini? IWAPO NDIO, ULIZIA: Je, unaweza kusema hii ilikuwa mara chache, mara nyingi, au wakati wote ulipokuwa hospitalini?* | YES, ALL OF THE TIME 4  YES, MOST OF THE TIME 3  YES, A FEW TIMES 2  NO, NEVER 1  DK/CAN'T REMEMBER 8  PREFER NOT TO ANSWER 9 |  |
| 1. Did you feel the health facility staff took the best care of you that they could when you received breastfeeding counseling during your antenatal visit(s) at Mbagathi in the last two weeks? IF YES, ASK: Would you say this was all of the time, most of the time, or a few times?   *Ulihisi muhudumu wa kituo cha afya aliyekupea ushauri wa kunyonyesha alikutunza bora zaidi alivyoweza?* *IWAPO NDIO, ULIZIA: Je, unaweza kusema hii ilikuwa mara chache, mara nyingi, au wakati wote ulipokuwa hospitalini?* | YES, ALL OF THE TIME 4  YES, MOST OF THE TIME 3  YES, A FEW TIMES 2  NO, NEVER 1  DK/CAN'T REMEMBER 8  PREFER NOT TO ANSWER 9 |  |
| 1. Were you in any way unhappy with how health facility staff treated you when you received breastfeeding counseling during your antenatal visit(s) at Mbagathi in the last two weeks? [DO NOT READ RESPONSE OPTIONS.] *Kwa namna yeyote unaweza kuwa hukufurahishwa na jinsi wahudumu wa kituo cha afya waliokupa ushauri wa kunyonyesha, walivyo kutendea?* [DO NOT READ RESPONSE OPTIONS.] | YES 1  NO 2 →  DK 8 →  PREFER NOT TO ANSWER 9 → | GO TO Q25  GO TO Q26GO TO Q26  GO TO Q26 |
| 1. Why were you unhappy with how the health facility staff treated you when you received breastfeeding counseling during your antenatal visit(s) at Mbagathi in the last two weeks? How did they treat you? *Kwa nini hukufurahishwa na jinsi wahudumu wa kituo cha afya waliokupa ushauri jinsi ya kunyonyesha walivyo kuchukulia?* |  |  |
| 1. Did the health facility staff treat you differently because of any personal attribute when you received breastfeeding counseling during your antenatal visit(s) at Mbagathi in the last two weeks? For instance, did they treat you differently because of your age, disability, marital status, number of children, education status, wealth, or something else? IF YES, ASK: Would you say this was all of the time, most of the time, or a few times? *Unaweza kusema kwamba wafanyi kazi wa kituo cha afya waliokupa ushauri wa kunyonyesha walikuchukulia tofauti kwa sababu ya sifa zozote ya kibinafsi kama vile umri wako, hali ya ndoa, idadi ya watoto, elimu, utajiri na mambo kama hayo?*   *IWAPO NDIO, ULIZIA: Je, unaweza kusema hii ilikuwa mara chache, mara nyingi, au wakati wote ulipokuwa hospitalini?* | YES, ALL OF THE TIME 4  YES, MOST OF THE TIME 3  YES, A FEW TIMES 2  NO, NEVER 1  DK/CAN'T REMEMBER 8  PREFER NOT TO ANSWER 9 |  |
| 1. Did the health facility staff physically mistreat you when you received breastfeeding counseling during your antenatal visit(s) at Mbagathi in the last two weeks? For instance, were you pushed, slapped, pinched, or physically mistreated in any other way? IF YES, ASK: Would you say this was all of the time, most of the time, or a few times? *Je, ulihisi ulichukuliwa visivyo, kwa mfano ukasukumwa, ukapigwa kofi, kufinywa, ama dhuluma zinginezo za kimwili haswa na mhudumu wa kituo cha afya aliyekupea ushauri wa kunyonyesha? IWAPO NDIO, ULIZIA: Je, unaweza kusema hii ilikuwa mara chache, mara nyingi, au wakati wote ulipokuwa hospitalini?* | YES, ALL OF THE TIME 4  YES, MOST OF THE TIME 3  YES, A FEW TIMES 2  NO, NEVER 1  DK/CAN'T REMEMBER 8  PREFER NOT TO ANSWER 9 |  |
| 1. Did the health facility staff verbally mistreat you when you received breastfeeding counseling during your antenatal visit(s) at Mbagathi in the last two weeks? For instance, were you shouted at, insulted, threatened, talked to rudely, or verbally mistreated in any other way? IF YES, ASK: Would you say this was all of the time, most of the time, or a few times? *Je, ulihisi yule mhudumu wa kituo cha afya aliyekupatia ushauri wa kunyonyesha alikufokea, karipia, kutusi, kutisha, alikuongelesha kwa madharau, ama alikudunisha kwa njia yoyote kwa matamshi? IWAPO NDIO, ULIZIA: Je, unaweza kusema hii ilikuwa mara chache, mara nyingi, au wakati wote ulipokuwa hospitalini?* | YES, ALL OF THE TIME 4  YES, MOST OF THE TIME 3  YES, A FEW TIMES 2  NO, NEVER 1  DK/CAN'T REMEMBER 8  PREFER NOT TO ANSWER 9 |  |
| 1. Do you think the breastfeeding counseling you received during your antenatal visit(s) at Mbagathi in the last two weeks was helpful? [DO NOT READ RESPONSE OPTIONS.]   *Je, unafikiria ushauri wa kunyonyesha uliopewa wakati wa ziara ya kliniki ya kabla ya kujifungua ulikuwa wa manufaa?* [DO NOT READ RESPONSE OPTIONS.] | YES 1 → NO 2 →  DK/CAN'T REMEMBER 8 →  PREFER NOT TO ANSWER 9 | GO TO Q29aGO TO Q29b  GO TO Q30  GO TO Q30 |
| 29(a) How was it helpful? [DO NOT READ RESPONSE OPTIONS. AS THE MOTHER RESPONDS, CHECK OFF THE RESPONSE(S) THAT MOST CLOSELY RESEMBLES WHAT SHE HAS SAID. FOR ALL OTHER RESPONSES NOT LISTED, RECORD HER RESPONSE UNDER “OTHER.” IF YOU ARE UNSURE, RECORD RESPONSES UNDER “OTHER.”] PROBE: Was it helpful in any other way? *Ilikusaidia vipi?* [DO NOT READ RESPONSE OPTIONS. AS THE MOTHER RESPONDS, CHECK OFF THE RESPONSE(S) THAT MOST CLOSELY RESEMBLES WHAT SHE HAS SAID. FOR ALL OTHER RESPONSES NOT LISTED, RECORD HER RESPONSE UNDER “OTHER”. IF YOU ARE UNSURE, RECORD RESPONSES UNDER “OTHER”.] *TAFUTA MAELEZO ZAIDI: Ilikusaidia kwa njia nyengineyo?* | THE HEALTH WORKER TAUGHT ME INFORMATION A  THE HEALTH WORKER HELPED ME GET MY BABY TO LATCH B  THE HEALTH WORKER HELPED ME POSITION MY BABY FOR BREASTFEEDING C  OTHER H  SPECIFY: _________ |  |
| 29(b) Why do you feel the breastfeeding counseling wasn’t helpful? [DO NOT READ RESPONSE OPTIONS. AS THE MOTHER RESPONDS, CHECK OFF THE RESPONSE(S) THAT MOST CLOSELY RESEMBLES WHAT SHE HAS SAID. FOR ALL OTHER RESPONSES NOT LISTED, RECORD HER RESPONSE UNDER “OTHER.” IF YOU ARE UNSURE, RECORD RESPONSES UNDER “OTHER.”] PROBE: Is there any other reason that you do not feel that it was helpful? *Kwa nini unahisi ushauri wa kunyonyesha haukuwa wa manufaa?* [DO NOT READ RESPONSE OPTIONS. AS THE MOTHER RESPONDS, CHECK OFF THE RESPONSE(S) THAT MOST CLOSELY RESEMBLES WHAT SHE HAS SAID. FOR ALL OTHER RESPONSES NOT LISTED, RECORD HER RESPONSE UNDER “OTHER.” IF YOU ARE UNSURE, RECORD RESPONSES UNDER “OTHER.”] *TAFUTA MAELEZO ZAIDI: Kuna sababu nyengine inayokufanya uhisi haukuwa na manufaa?* | I ALREADY KNEW EVERYTHING I NEEDED TO KNOW L  THE HEALTH WORKER DID NOT KNOW WHAT THEY WERE DOING M  THE HEALTH WORKER DID NOT EXPLAIN THINGS WELL N  I WAS NOT ABLE TO LATCH AND/OR POSITION MY BABY WELL O  OTHER S  SPECIFY: _________ |  |
| 1. Overall, were you satisfied or dissatisfied with the breastfeeding counseling you received during your antenatal visit(s) at Mbagathi in the last two weeks? IF SATISFIED, PROBE: Somewhat or very satisfied? IF DISSATISFIED, PROBE: Were you very dissatisfied, somewhat dissatisfied? *Kwa ujumla, uliridhika vipi na ule ushauri wakunyonyesha uliopewa wakati wa ziara ya kliniki ya kabla ya kujifungua? KAMA ULIRIDHIKA, NI KWA KIASI KIPI: uliridhika kabisa ama uliridhika kwa kadri? KAMA HUJARIDHIKA, NI KWA KIASI KIPI: Sijaridhika kwa kadri na sijaridhika kabisa?* | VERY SATISFIED 1  SOMEWHAT SATISFIED 2  NEUTRAL 3  SOMEWHAT DISSATISFIED 4  VERY DISSATISFIED 5  DK/CAN'T REMEMBER 8  PREFER NOT TO ANSWER 9 |  |
| Is there anything else you would like to tell me about today about the breastfeeding counseling you received during your antenatal visit(s) at Mbagathi in the last two weeks?*Na kuna jambo lingine ungependa kuniambia kuhusu ushauri wa kunyonyesha?* RECORD RESPONSE. IF NOTHING ELSE, RECORD “NOTHING ELSE.” |  |  |

Thank you so much for your time! *Asante sana kwa wakati wako!*
